# Towards the Synthesis of Pyoverdines: Preparation and Reactivity of the *N*-Formylhydroxyornithine Residue

**DOI:** 10.3390/molecules31121988

**Published:** 2026-06-06

**Authors:** Tianzhu Zhang, Albert Bolhuis, Ian M. Eggleston

**Affiliations:** Department of Life Sciences, University of Bath, Bath BA2 7AY, UK; zhangtianzhu1996@gmail.com (T.Z.); ab366@bath.ac.uk (A.B.)

**Keywords:** iron, siderophore, cyclic peptide, *N*-oxidation, protecting group, SPPS

## Abstract

The Gram-negative bacterium *Pseudomonas aeruginosa* produces a family of peptide siderophores called pyoverdines that play a vital role in the mechanisms by which it acquires iron from the environment. A key component of various pyoverdines is the presence of one or more copies of L-δ-*N*-formyl-δ-*N*-hydroxyornithine (fOHOrn) as an iron-binding residue. In this study, we have developed an improved preparation of a derivative of fOHOrn that is suitable for use in solid-phase peptide synthesis, incorporating a novel *N*-oxidation protocol and a mild final deprotection with HCl/hexafluoroisopropanol (HFIP) that circumvents the unexpected deformylation of the fOHOrn side chain under acidic conditions. We have also devised a synthesis of the cyclic peptide component of pyoverdine D exploiting a selective side-chain deprotection strategy with HCl/HFIP that allows the application of readily available amino acids with standard *tert*-butyl side-chain protection and which facilitates the cyclisation step. These innovations open the way towards the convergent preparation of various pyoverdines and also other natural products that contain fOHOrn residues.

## 1. Introduction

**Figure 1:**
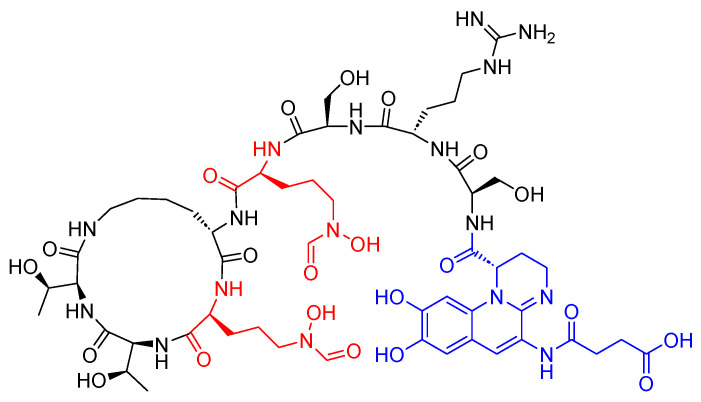
Structure of pyoverdine D. The fOHOrn residues are highlighted in red; the chromophore unit is shown in blue.

Antimicrobial resistance (AMR) is a major ongoing global health challenge, with deaths associated with AMR currently estimated to exceed 8 million per year by 2050 [1]. There is thus an urgent need to explore novel antibacterial strategies and to develop new ways to target pathogens that are becoming resistant to conventional antibiotics [2,3]. In this context, the mechanisms by which bacteria acquire iron from their hosts have attracted major research interest, since iron is essential to the growth and development of nearly all microorganisms [4,5], including the formation of biofilms, which are a major source of antibiotic resistance [6]. Bacteria have evolved to produce a range of molecules called siderophores, which have a high affinity for ferric iron and are secreted into the host environment to capture iron, whereupon the complex is recognized and transported into the bacterial cell [7]. *Pseudomonas aeruginosa*, a Gram-negative bacterium responsible for various life-threatening infections, produces primarily two types of siderophore, the most important of which is a family of peptides known collectively as pyoverdines [8]. Over 100 pyoverdines have been identified from different *Pseudomonas* strains, each consisting of a linear or partly cyclised peptide unit containing two metal-chelating amino acid residues, as well as a fluorescent, metal-binding dihydroxyquinoline chromophore, with a variable side-chain substituent [9]. Pyoverdine D (Figure 1), a cyclic peptide of the so-called pyoverdine class I (PVDI), is an important example, and like many other pyoverdines, it contains the non-proteinogenic amino acid L-δ-*N*-formyl-δ-*N*-hydroxyornithine (fOHOrn) as an iron-binding residue. Pyoverdine D contains two fOHOrn residues, and this unit also plays a key role in the iron-binding properties of various other siderophores from *Pseudomonas* and other bacterial species [10,11].

An attractive approach to improve the efficacy of existing antibiotics is to target them to otherwise resistant bacteria such as *P. aeruginosa*, by linking them to their endogenous siderophores such as pyoverdines to then exploit the bacteria’s own transporters for the delivery of these therapeutics [12,13]. This so-called Trojan Horse strategy has received considerable attention, and a range of siderophore antibiotic conjugates (SACs) have now undergone clinical trials [14]. Pyoverdine D has attracted interest not only for the development of SACs, but also as a tool for bioimaging [15] and biosensing applications [16]. So far, there have been few reports on the preparation of pyoverdines that would be applicable for a wide range of analogues for structure-activity studies and also the development of conjugates involving diverse antibiotics or other conjugates with multifunctional biomolecules. Although a chemical synthesis of pyoverdine D has been reported by Maschiach and Meijler [17], it utilizes some relatively costly protected amino acids as part of the required multi-level orthogonal protection strategy and also involves coupling of the valuable pyoverdine chromophore to the peptide backbone on solid phase as part of the approach, which may therefore limit the scale of preparations. A recent report by Puja et al. [18] on a biosynthetic route to a clickable pyoverdine has also been presented, but such an approach is not yet suitable for the production of significant quantities of SACs for detailed biological studies. As part of our own interest [19] in the application of pyoverdine derivatives as tools for biomedical research, we have therefore been focusing on the development of fully convergent and scalable synthetic pathways that are compatible with both readily available protected amino acid derivatives and the incorporation of the non-proteinogenic metal-binding residues and diverse chromophore units. In this context, we set out to develop an effective synthesis of a suitably protected form of the peptide component of pyoverdine D, which could then ultimately be coupled in solution with the natural chromophore. We describe here an improved, practical synthesis of an fOHOrn derivative suitable for use in Fmoc-solid-phase peptide synthesis (SPPS) and some previously unreported features of the reactivity of this novel amino acid that are relevant to its incorporation into complex peptide structures.

## 2. Results and Discussion

### 2.1. Synthesis of Fmoc-Protected fOHOrn

**Scheme 1:**
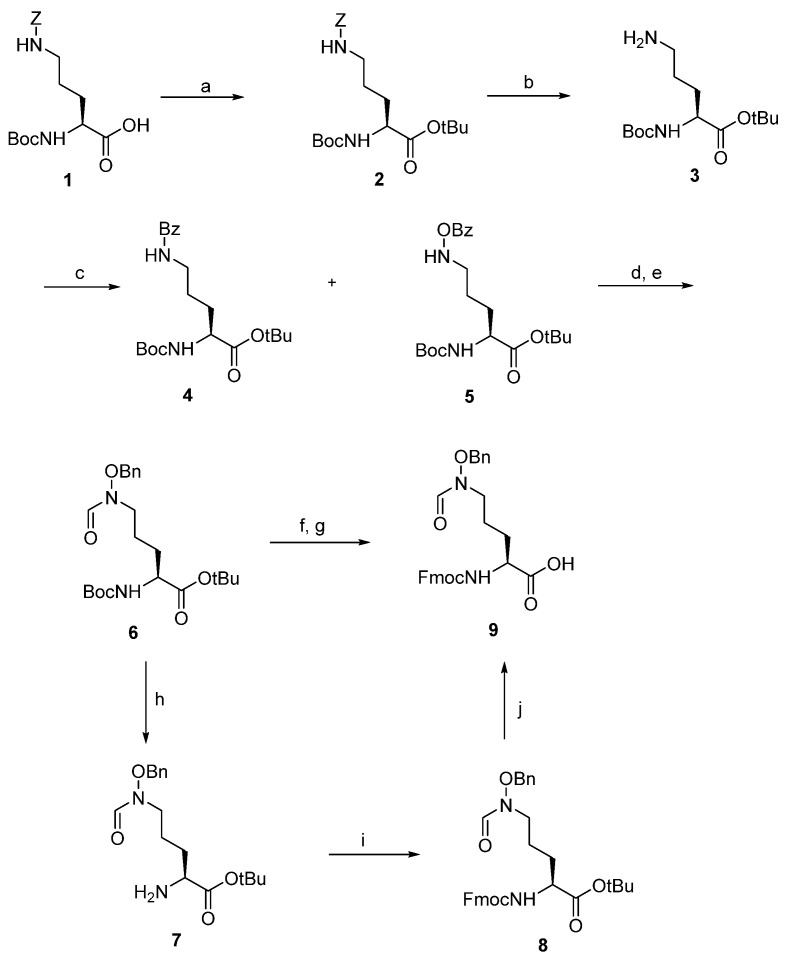
Synthesis of Fmoc-fOHOrn derivative **9** from Boc-Orn(Z)-OH. *Reagents and conditions:* (a) Boc_2_O, DMAP, *tert*-butanol, 30 °C, 14 h, 92%. (b) Pd/C, MeOH, H_2_, rt, 2 h, 83%. (c) Cs_2_CO_3_, BPO, DCM, rt, 2 h, **4**: 11%; **5**: 52%. (d) EDC·HCl, HCO_2_H, DCM, 0 °C to rt, 4 h. (e) MeOH, DIEA, BnBr, rt, 16 h, 81% over two steps. (f) 50% TFA/DCM, rt, 3.5 h. (g) Fmoc-OSu, NaHCO_3_, THF, H_2_O, rt, 23 h, 25% over two steps. (h) 4 M HCl in dioxane, 0 °C to rt, 30 min. (i) Fmoc-Cl, DIEA, DCM, rt, 16 h. (j) 0.1 M HCl/HFIP, rt, 4 h, 70% over three steps.

Scheme 1 shows our approach to a Fmoc-protected fOHOrn derivative, suitable for use in SPPS. Mashiach and Meijler [17] have previously described the preparation of this Fmoc-protected derivative of fOHOrn following the strategy of Fennell and Miller [20], by sequential *N*-oxidation and formylation of the side-chain function of a suitably protected ornithine derivative. We initially planned to adopt this strategy directly, and starting from Boc-Orn(Z)-OH **1**, conversion to the *tert*-butyl ester **2** was achieved by treatment of **1** with Boc_2_O and catalytic DMAP in *tert*-butanol according to the method of Litzinger et al. [21]. This gave **2** in excellent yield when the reaction time was restricted to 14 h. On a large scale, extending the reaction time further to promote full consumption of starting material led to the formation of some doubly *N^δ^*-acylated material, which then significantly complicated purification and decreased the isolated yield of **2**. After standard hydrogenolysis, **3** with a free amine side chain was obtained in 76% yield over the two steps, ready to carry out the key side-chain oxidation. We then initially attempted to apply the procedure of Fennell and Miller [20], as utilized by Mashiach and Meyler [17], wherein Boc-Orn-OtBu **3** was initially treated with dibenzoylperoxide (BPO) in a two-phase system of NaHCO_3_/Na_2_CO_3_ buffer solution and DCM (pH = 10.5). However, after work-up and isolation by chromatography, only the undesired *N*-benzoylated derivative **4** was obtained in 14% yield. Significantly, it was also observed that the pH of the final reaction mixture had fallen to below 9. Competing *N*-benzoylation has been previously observed when various amines are treated under similar conditions with BPO, typically requiring careful pH control to favour the desired *N*-O linked products [22,23]. In search of an operationally simpler alternative, we were therefore attracted by the study of Banerjee and Yamamoto, which investigated the optimization of the BPO oxidation reaction of various primary amines and diamines by comparing different bases and the effect of H_2_O on the overall yield and ratio of O to *N*-acylated products [24]. Inspired by their approach, **3** was treated with Cs_2_CO_3_ and 75% *w*/*w* BPO (remaining 25% H_2_O) to give the desired product **5** in 52% yield and **4** in 11% yield. This result compares very favourably with the outcome reported by Mashiach and Meyler for the oxidation of **3** under biphasic conditions (55% yield of **5**) [17], and in our hands the Cs_2_CO_3_ proved to be both robust and reproducible, removing the need for strict pH monitoring [22]. Consistent with Banerjee and Yamamoto’s findings [24], we also observed that optimum results were obtained using dry DCM in the presence of the fixed quantity of H_2_O present in the oxidant (25% *w*/*w*). The origin of the selectivity in this oxidation presumably arises from preferential complexation of BPO with the Cs+ ion, as other metal carbonates are reported to give significantly lower conversions and poorer selectivities [24]. To our knowledge, this is the first application of the Cs_2_CO_3_-mediated BPO oxidation in an amino acid system and thus provides a potential alternative for this key step in the preparation of other *N*-hydroxyornithine and *N*-hydroxylysine systems.

Efficient formylation of **5** was achieved by treatment with EDC (4 eq) and formic acid (4 eq) in DCM for 4 h. One-pot removal of the benzoyl group and reprotection via the benzyl hydroxamate was then achieved to give **6** in 81% yield over two steps by treatment with DIEA and benzyl bromide in anhydrous MeOH. We then expected to obtain the final ornithine building block for SPPS by simple treatment of **6** with 50% TFA/DCM to remove Boc and tBu protection, followed by re-protection with Fmoc, as described by Mashiach and Meijler [17]. Removal of both the Boc and tert-butyl protecting groups of **6** was, however, only complete as judged by HPLC after 3.5 h at room temperature, and following reaction with Fmoc-OSu in aq NaHCO_3_/THF, the desired **9** was only isolated in pure form in 25% yield (two steps) after careful chromatography. Significantly, mass spectrometry analysis of the crude material also revealed the presence of *N*-deformylated material, presumably resulting from the prolonged exposure of **6** to strongly acidic conditions. *N*-deformylation of α-amino acids and peptides under acidic conditions has been reported [25], but this normally occurs only slowly at room temperature with dilute mineral acids, suggesting that the alkoxyamide moiety of compounds such as **6** may be unusually sensitive, possibly due to the inductive effect of the oxygen substituent disturbing the normal amide resonance within the *N*-formyl group [26].

To address this issue, and to simplify the isolation of **9**, the conversion from **6** was separated into three selective and high-yielding steps. Firstly, selective removal of the Boc group in the presence of the *tert*-butyl protection to give **7** was effected with 4 M HCl in dioxane at low temperature [27]. Introduction of the Fmoc group could then be efficiently achieved with Fmoc-Cl to give the fully protected derivative **8** in 70% yield, without risk of formation of any dipeptide by-products [28]. Purification by column chromatography at this stage also avoided the previously challenging purification of the more polar **9**. Crucially, the final removal of the tert-butyl ester protecting group could be achieved using 0.1 M HCl in HFIP for 4 h to give **9** in quantitative yield without affecting the integrity of the fOHOrn residue and also with no need for any additional purifications. These TFA-free conditions have been previously found to give clean and rapid removal of a range of acid-labile protecting groups commonly used in peptide synthesis, including *tert*-butyl esters and ethers, Boc, trityl, and Pbf [29], and have proved to be effective for deprotections in other sensitive amino acid substrates [30]. Notably, when the final deprotection of **8** was attempted instead with TFA/DCM, the reaction was again incomplete after 3 h, and a complicated HPLC chromatogram was observed when the reaction was extended to 14 h. In summary, compound **9,** prepared by our three-step method using HCl/HFIP, was isolated in a superior overall yield to the previously reported two-step conversion of **6** to **9** (70% from **6** vs. 50% [17]) without any loss of efficiency, and it could be employed directly in SPPS following the final deprotection step.

### 2.2. Synthesis of the Peptide Unit of Pyoverdine D

**Scheme 2:**
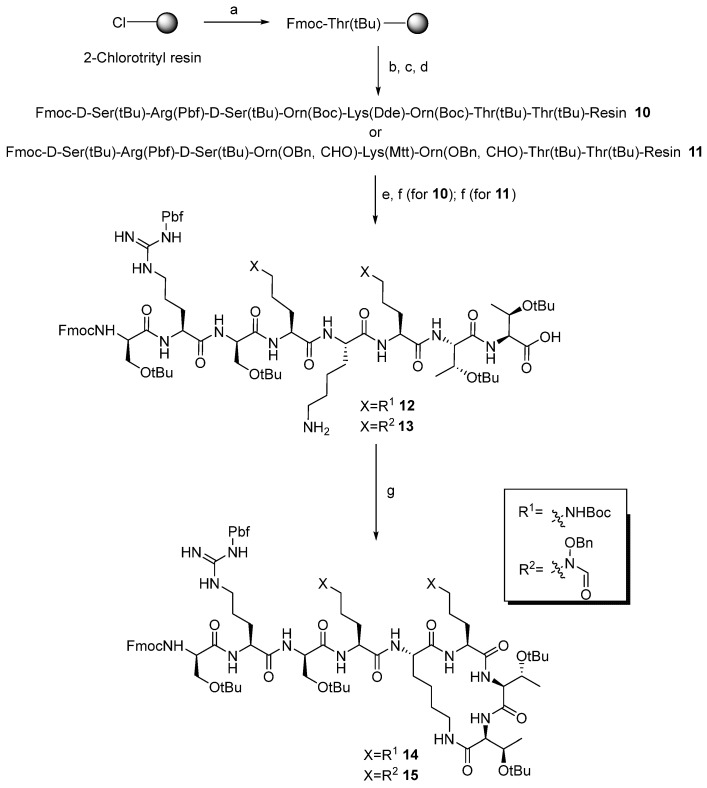
Solid-phase peptide synthesis of linear fOHOrn and Orn-containing peptides **12** and **13** and attempted cyclisation to pyoverdine precursors **14** and **15**. *Reagents and conditions:* (a) Fmoc-Thr(tBu)-OH, DIEA, DCM. (b) 20% piperidine/DMF. (c) PyBOP, DIEA, DMF, Fmoc-Xaa-OH or HATU, DIEA, DMF for **9**, HATU, TMP, DMF for Fmoc-D-Ser(tBu)-OH. (d) repeat step b and c. (e) NH_2_OH, imidazole, NMP/DCM. (f) 1% TFA/DCM. (g) HATU, DIEA, DMF, or PyBOP, DIEA, DMF.

Scheme 2 outlines our initial approaches towards an orthogonally protected form of the pyoverdine D cyclic peptide, suitable for eventual elaboration to the natural siderophore by coupling with the relevant chromophore or conjugation with other chelating moieties. In order to conserve the valuable intermediate **9**, a linear peptide precursor **10** incorporating Orn(Boc) residues was first assembled on 2-chlorotrityl resin [31] according to standard Fmoc protocols [32] with PyBOP as a coupling agent, except for the coupling of Fmoc-D-Ser(tBu)-OH, where HATU (2 eq) and 2,4,6-trimethylpyridine (TMP, 4 eq) were used to avoid possible epimerisation, as recommended by Di Fenza et al. [33]. In parallel, peptide resin **11,** in which the fOHOrn residues were incorporated, was also assembled in a similar fashion, but using HATU and DIEA for coupling of **9**.

For the incorporation of the Lys residue in the linear sequences, we initially anticipated using Lys(Dde) in order to provide an extra level of orthogonality for future potential investigations on the synthesis of pyoverdine conjugates. Thus, in the case of **10,** where this was employed, selective deprotection of Lys(Dde) was accomplished on resin with NH_2_OH·HCl/imidazole to avoid Fmoc removal [34], and this was then followed by treatment with 1% TFA/DCM to release the linear peptide **12** ready for cyclisation. However, preliminary experiments with **8** (see Supplementary Material) subsequently revealed that the fOHOrn side chain was unstable towards NH_2_OH·HCl/imidazole, resulting in the loss of the formyl unit. Thus, for peptide resin **11,** Lys(Mtt) was employed instead, and cleavage from the resin and concomitant removal of Mtt was achieved directly by treatment with 1% TFA/DCM to give the corresponding linear peptide **13**.

Cyclisation of **13** was first attempted using similar conditions to those applied by Mashiach and Meijler for a closely related peptide in their synthesis of pyoverdine D [17]. Upon treatment of **13** with HATU (10 eq) and DIEA (10 eq) under high dilution after 14 h, however, the conversion to **15** was incomplete as judged by HPLC, and upon prolonging the reaction time to 48 h, a complicated mixture was observed, generating multiple species with similar or shorter retention times to those of the starting material (see Supplementary Material, Figure S1). Surprisingly, when peptide **12** with Orn(Boc) was exposed to the same cyclisation conditions, a relatively clean conversion to the corresponding cyclic peptide **14** was observed within 2 h, which was further improved when HATU was replaced with PyBOP (Supplementary Material, Figure S2). Applying the same conditions to **13,** however, gave only a modest improvement, with partial conversion to **15** being observed after 14 h (Supplementary Material, Figure S3). A better outcome on a trial scale was in fact obtained when only a small excess of PyBOP was employed, suggesting that the previously observed side products might be associated with prolonged exposure of **13** to the excess coupling reagents and base. Under such conditions with limited coupling agent (1.2 eq PyBOP, 2 eq DIEA), **15** was successfully isolated in 41% yield after HPLC. For the attempted cyclisations with HATU, one possible competing reaction is the guanidinylation of the peptide *N*-terminus through direct reaction with the coupling agent itself [35]; however, analysis of the crude reaction products by mass spectrometry did not reveal the formation of any such products.

**Scheme 3:**
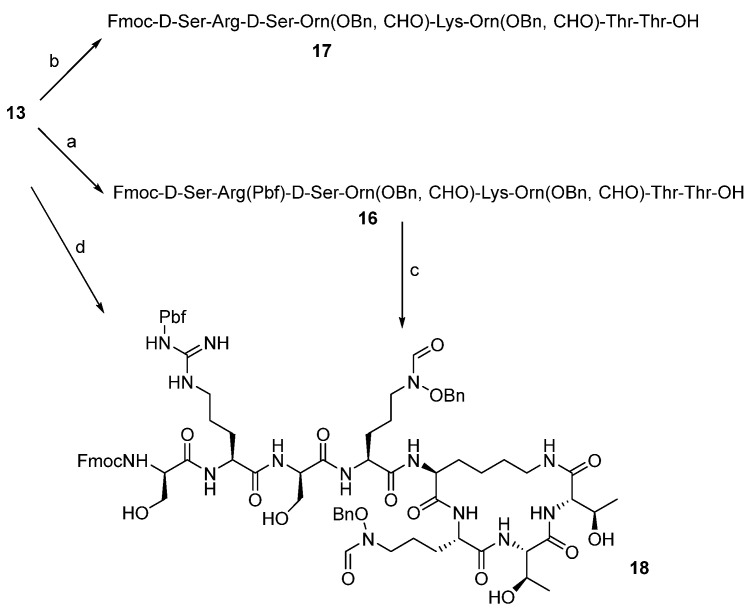
Selective deprotection of **13** and cyclization of partially protected **16** to **18**. *Reagents and conditions:* (a) 0.1 M HCl in HFIP, 10 min, 92%; (b) 0.1 M HCl in HFIP, 60 min, 54%; (c) PyBOP, DIEA, DMF, 40%; (d) step a, then PyBOP, DIEA, DMF, 70% over two steps for **18**.

We speculated that the incomplete and slow cyclisation of **13** could be associated with steric hindrance around the side-chain protected C-terminal residue (Thr(tBu)), since it is well-documented that the efficiency of peptide cyclisations may be impaired if the site of ring closure is sterically encumbered, for instance by β-branched amino acids [36,37]. In Mashiach and Meyler’s work, cyclisation was achieved without protection of Ser and Thr residues, which were introduced into the linear precursor via more costly trityl-protected amino acid derivatives, the side-chain protection being removed upon cleavage from the solid phase prior to the cyclisation step [17]. To test our hypothesis and drawing on our discovery of the compatibility of the fOHOrn residue with HCl/HFIP, partial deprotection of **13** to **16** was attempted (Scheme 3). Gratifyingly, when **13** was treated with 0.1 M HCl in HFIP for 10 min, the desired partially protected peptide was isolated in 93% yield with removal of *tert*-butyl protection at Ser and Thr residues only. Removal of Pbf protection to give **17** could also be achieved effectively upon extending the exposure time to 1 h (see Supplementary Material Figure S4), again without compromising the integrity of the sensitive fOHOrn residues. Cyclisation of **16** was then attempted with 3 eq PyBOP in DMF. Consistent with our hypothesis, in the absence of the bulky-OtBu substituent at the C-terminal Thr, cyclisation of **16** proceeded cleanly to generate the desired cyclic peptide **18** in as little as 30 min (see Supplementary Material Figure S5) and in 40% isolated yield after preparative HPLC.

Although we cannot exclude the possibility that the cyclisation issue may also be associated with the protected peptide **13** adopting a conformation that is less favourable to macrolactamisation [37] than **16**, which is resolved by partial deprotection, the overall approach we adopted has a valuable practical implication. That is, it provides a way of utilizing standard *tert*-butyl side-chain protection for Ser and Thr residues, avoiding other potentially less economical and more sensitive protected derivatives. When carrying out the partial deprotection of **13**, to avoid any unwanted deformylation of fOHOrn, it was found to be necessary to rigorously remove residual acid from the partially deprotected linear peptide if it was not to be utilized immediately. However, using **16** directly after deprotection was also found to be effective when crude **16** was immediately dissolved in anhydrous DMF and neutralized with DIEA prior to addition of PyBOP, cyclic peptide **18** could be obtained in an enhanced 70% yield over two steps from the initial linear fOHOrn precursor **13**.

In summary, a key Fmoc-protected fOHOrn building block **9** for pyoverdine synthesis was prepared by our optimized approach in 22% yield over seven steps, starting from readily available Boc-L-Orn(Z)-OH **1**. This improved synthetic strategy using the efficient Cs_2_CO_3_/BPO oxidation and mild HCl/HFIP deprotection should also be applicable for the preparation of other formyl- or acetyl-hydroxyornithine or hydroxylysine derivatives, which occur widely in peptide siderophores [9]. Our research has also revealed an unexpected acid sensitivity for the side chain of fOHOrn, indicating that the synthesis of such peptides may require careful planning of protecting group strategies and the choice of final deprotection conditions. In this work, a protecting group strategy was identified based on readily available Fmoc-amino acid building blocks, and by exploiting again HCl/HFIP conditions, the preparation of the cyclic peptide unit of pyoverdine D could be facilitated. Such application of HCl/HFIP for selective acidic deprotections within intact peptides should warrant further study for the preparation of partially protected peptides and also the development of alternative and more environmentally friendly deprotection strategies in peptide synthesis, particularly with highly sensitive substrates [30]. With reliable methodology in place for the preparation of fOHOrn peptides such as **18**, the scene is now set for both a convergent and practical synthesis of pyoverdine D and also a range of pyoverdine conjugates whose biological potential may be explored. Further studies in this area are now ongoing in our laboratories and will be reported in due course.

## 3. Experimental Section

### 3.1. Materials

All reactions were carried out using oven-dried glassware. All reagents and solvents were purchased from Sigma-Aldrich (St. Louis, MO, USA), Fluorochem (Glossop, UK), Fisher (Waltham, MA, USA), Alfa Aesar (Ward Hill, MA, USA), Merck (Darmstadt, Germany), Apollo (Osaka, Japan) and Acros Organics (Geel, Belgium). Anhydrous DCM was dried by distillation under argon. Peptide-grade DMF was purchased from Supelco (Bellefonte, PA, USA). Thin-layer chromatography (TLC) was performed on silica gel 60 F_254_ pre-coated on aluminum sheets (Merck). Flash chromatography was performed on silica gel 60 (35–70 micron) from Fisher.

Mass spectrometry was performed using an Agilent QTOF 6545 with Jetstream ESI spray source coupled to an Agilent 1260 Infinity II Quat pump HPLC (Agilent Technologies, Santa Clara, CA, USA). ^1^H and ^13^C NMR were obtained using Bruker Avance III 400 MHz and 500 MHz instruments, ^13^C NMR data is either 100 MHz or 125 MHz (Bruker, Billerica, MA, USA). Chemical shift values stated are in parts per million (ppm) and coupling constant values (*J*) are given in Hertz (Hz). Optical rotations were measured using an Optical Activity Ltd. (Bury, UK). AA-10 Series Automatic Polarimeter, with a path length of 1 dm, and with concentration (c) quoted in g/100 mL. Analytical HPLC (System A) was carried out on a Dionex Ultimate 3000 HPLC instrument (Sunnyvale, CA, USA) using a Phenomenex Gemini 5 μm C-18 110A column with a flow rate of 1 mL/min at 35 °C (Phenomenex, Torrance, CA, USA). UV detection was carried out at 214, 220, 254 and 280 nm. The solvent system for the mobile phase contained either 0.1% TFA in H_2_O (A) or 0.1% TFA in MeCN (B). Analytical HPLC (System B) was carried out on a Dionex HPLC system using a Phenomenex Gemini 5 μm C-18 110A column with a flow rate of 1 mL/min at 35 °C. UV detection was carried out at one of 214, 220, 254 and 280 nm. The solvent system for the mobile phase contained either 0.1% TFA in H_2_O (A) or 0.1% TFA in MeCN (B). Preparative RP-HPLC was performed on a Dionex HPLC system equipped with a Phenomenex Gemini 5 µm C-18 (250 × 10 mm) column with a flow rate of 22.5 mL/min. Mobile phase A was 0.1% TFA in H_2_O and mobile phase B was 0.1% TFA in MeCN. Gradient 1 was *T* = 0 min, *B* = 5%; *T* = 10 min, *B* = 95%; *T* = 15 min, *B* = 95%; *T* = 15.1 min, B = 5%; *T* = 18.1 min, *B* = 5%. Gradient 2 was *T* = 0 min, *B* = 5%; *T* = 25 min, *B* = 95%; *T* = 35 min, *B* = 95%; *T* = 35.1 min, *B* = 5%. The completeness of solid-phase coupling and Fmoc deprotection was determined by the Kaiser test [38]. A few beads were placed in a test tube, and 3 drops of the following prepared solutions were added: (1) 50 mg/mL ninhydrin in EtOH, (2) 40 mg/mL phenol in EtOH, (3) 0.02 mM KCN in pyridine. The tube was mixed and heated by a heat gun for 2–3 min. A dark-blue colouration of the bead indicated the presence of a free amino function.

### 3.2. Synthesis of fOHOrn Derivatives

*tert-Butyl-(S)-5-(((benzyloxy)carbonyl)amino)-2-((tert-butoxycarbonyl)amino)pentanoate (***2***)* [21]

A solution of Boc-L-Orn(Z)-OH (**1**) (1.00 g, 2.73 mmol) with Boc_2_O (960 mg, 7.33 mmol) and DMAP (0.10 g, 0.82 mmol) in *tert*-butanol (10 mL) was stirred at 30 °C. After 14 h, the solvent was removed under reduced pressure, and the residue was subjected to chromatography (1:4 to 1:2 EtOAc: petroleum ether) to give Boc-L-Orn(Z)-OtBu (**2**) (1.06 g, 92%) as an oil. Rf = 0.4 (19:1 DCM: Acetone); ^1^H NMR (CDCl_3_, 400 MHz): δ 1.46 (9H, s, (CH_3_)_3_), 1.48 (9H, s, (CH_3_)_3_), 1.55–1.67 (3H, m, β-CH_2_, γ-CH_2_), 1.80–1.86 (1H, m, β-CH_2_, γ-CH_2_), 3.21–3.26 (2H, m, δ-CH_2_), 4.16–4.20 (1H, m, α-CH), 4.88–4.92 (1H, m, NH), 5.07–5.08 (1H, m, NH), 5.11 (2H, s, CH_2_), 7.33–7.38 (5H, m, 5 × Ar-CH); ^13^C NMR (CDCl_3_, 100 MHz): δ 171.7, 156.4, 155.4, 136.6, 128.6, 128.5, 128.1, 82.0, 79.7, 66.6, 53.5, 40.6, 30.3, 28.3, 28.0, 25.8; found (ESI^+^) 423.2496 [M + H]^+^, C_22_H_35_N_2_O_6_ requires 423.2490.

On a larger scale (e.g., 13.6 mmol), prolonged reaction led to the formation of material incorporating an additional Boc group, found (ESI^+^) 523.3011 [M + Na]^+^, C_21_H_32_N_2_O_5_Na requires 523.3014.

*tert-Butyl (S)-5-amino-2-((tert-butoxycarbonyl)amino)pentanoate (***3***)*

A suspension of Boc-L-Orn(Z)-OtBu (**2**) (2.30 g, 5.45 mmol) and 10% Pd/C (460 mg) in MeOH (20 mL) was stirred at room temperature for 2 h under H_2_. The crude mixture was filtered through Celite, and the solvent was evaporated to give Boc-Orn-OtBu (**3**) (1.3 g, 83%), which was used immediately. ^1^H NMR (CDCl_3_, 500 MHz): δ 1.46 (9H, s, (CH_3_)_3_), 1.50 (9H, s, (CH_3_)_3_), 1.88–1.93 (4H, m, β-CH_2_, γ-CH_2_), 3.06–3.10 (2H, m, δ-CH_2_), 4.15–4.18 (1H, m, α-CH), 5.36–5.38 (1H, m, NH); ^13^C NMR (CDCl_3_, 125 MHz): δ 171.7, 155.6, 82.1, 79.7, 53.6, 40.5, 30.1, 28.4, 28.0, 26.1. ^1^H NMR and ^13^C NMR data were consistent with those reported by Litzinger et al. [21].

*tert-Butyl (S)-5-benzamido-2-((tert-butoxycarbonyl)amino)pentanoate (***4***) and tert-Butyl (S)-5-((benzoyloxy)amino)-2-((tert-butoxycarbonyl)amino)pentanoate (***5***)*

*Method A* [17]: NaHCO_3_ (152 mg, 1.80 mmol) and Na_2_CO_3_ (860 mg, 8.18 mmol) were dissolved in distilled H_2_O (100 mL) to prepare a NaHCO_3_/Na_2_CO_3_ buffer solution (pH = 10.5). Boc-L-Orn-OtBu (**3**) (420 mg, 1.40 mmol) was dissolved in DCM (5 mL), and the resulting solution was diluted with NaHCO_3_/Na_2_CO_3_ buffer solution (5 mL). BPO (364 mg, 1.50 mmol) was added to the biphasic solution, and the resulting solution was stirred vigorously at room temperature for 4.5 h. The reaction solution was separated, and the aqueous phase was extracted with DCM (3 × 10 mL), and the combined organic layers were washed with 10% Na_2_S_2_O_3_ (2 × 30 mL), dried over MgSO_4_, filtered, and the solvent was evaporated to give the crude product. TLC of the crude product indicated multiple products. The crude material was subjected to chromatography (1:4 to 1:2 EtOAc: petroleum ether) to give (**4**) (100 mg, 14%) as an oil.

*Method B* [24]: A suspension of Cs_2_CO_3_ (1.97 g, 8.10 mmol) and benzoyl peroxide (1.33 g, 5.50 mmol) in distilled DCM (12 mL) was stirred at room temperature under N_2_. After 2 h, a solution of Boc-L-Orn-OtBu (**3**) (800 mg, 2.70 mmol) in distilled DCM (5 mL) was added, and the mixture was further stirred at room temperature. After 16 h, H_2_O (10 mL) was added, and the resulting solution was stirred for 5 min and then extracted with DCM (20 mL). The organic layer was washed with brine (2 × 25 mL), dried over MgSO_4_, and the solvent was evaporated to give the crude product. Purification by column chromatography (1:4 to 1:2 EtOAc: petroleum ether) gave (**4**) (120 mg, 11%) and (**5**) (610 mg, 52%).

*Data for* (**4**): Rf = 0.51 (1:1 EtOAc: petroleum ether); ^1^H NMR (CDCl_3_, 400 MHz): δ 1.47 (9H, s, (CH_3_)_3_), 1.49 (9H, s, (CH_3_)_3_), 1.72–1.75 (3H, m, β-CH_2_, γ-CH_2_), 1.90–1.91 (H, m, β-CH_2_, γ-CH_2_), 3.47–3.65 (2H, m, δ-CH_2_), 4.22–4.24 (1H, m, α-CH), 5.21–5.23 (1H, m, NH), 6.71 (1H, s, NH), 7.43–7.53 (3H, m, 3 × Ar-CH), 7.83 (2H, d, *J* 7.4, 2 × Ar-CH); ^13^C NMR (CDCl_3_, 100 MHz): δ 171.7, 167.6, 155.6, 134.6, 131.3, 128.5, 127.0, 82.2, 79.9, 53.3, 39.3, 30.8, 28.3, 28.0, 25.2; found (ESI^+^) 415.2204 [M + Na]^+^, C_21_H_32_N_2_O_5_Na requires 415.2203.

*Data for* (**5**): Rf = 0.69 (1:1 EtOAc: petroleum ether); ^1^H NMR (CDCl_3_, 400 MHz): δ 1.45 (9H, s, (CH_3_)_3_), 1.47 (9H, s, (CH_3_)_3_), 1.62–1.77 (4H, m, β-CH_2_, γ-CH_2_), 1.92–1.99 (4H, m, β-CH_2_, γ-CH_2_), 3.17–3.20 (2H, m, δ-CH_2_), 4.21–4.24 (1H, m, α-CH), 5.11–5.12 (1H, d, *J* 8.0, NH), 7.46–7.50 (2H, m, 2 × Ar-CH), 7.69–7.63 (1H, m, Ar-CH), 7.92 (1H, s, NH), 8.02–8.04 (2H, m, 2 × Ar-CH); ^13^C NMR (CDCl_3_, 100 MHz): δ 171.7, 166.9, 155.4, 133.4, 129.3, 128.5, 128.3, 82.0, 79.7, 53.7, 52.1, 30.5, 28.3, 28.0, 23.2; found (ESI^+^) 409.2333 [M + H]^+^, C_21_H_33_N_2_O_6_ requires 409.2333.

*tert-Butyl (S)-5-(N-(benzyloxy)acetamido)-2-((tert-butoxycarbonyl)amino)pentanoate (***6***)* [17]

A solution of formic acid (0.55 mL, 14.7 mmol) and EDC hydrochloride (2.81 g, 14.7 mmol) in distilled DCM (8 mL) was stirred at 0 °C. After 15 min, a solution of Boc-Orn(OBz)-O^t^Bu (**5**) (1.5 g, 3.67 mmol) in distilled DCM (10 mL) was added, and the mixture was further stirred at 0 °C for 1 h and at room temperature. After 4 h, the solvent was removed under reduced pressure and the residue **7** was dissolved in EtOAc (50 mL). The resulting solution was washed with H_2_O (2 × 75 mL) and brine (2 × 75 mL) and dried over MgSO_4_. The solvent was evaporated, and the crude amide was taken on without further purification. A solution of the above crude material in anhydrous MeOH (80 mL) was prepared at room temperature. A solution of DIEA (1.27 mL, 7.34 mmol) and BnBr (1.30 mL, 11.01 mmol) was added, and the mixture was stirred at room temperature for 16 h under N_2_. The MeOH was evaporated off, and the residue was dissolved in EtOAc (80 mL) and cooled to 0 °C for 15 min. The white precipitate was filtered off, and the filtrate was washed with H_2_O (2 × 80 mL), brine (2 × 80 mL). The organic extracts were dried over MgSO_4_. The solvent was removed under reduced pressure, and the residue was subjected to chromatography (1:6 to 1:4 EtOAc: petroleum ether) to give (**6**) (1.25 g, 81%) as an oil. Rf = 0.42 (1:1 EtOAc: petroleum ether); HPLC (System A, Gradient 1) R_t_ = 10.57 min; ^1^H NMR (CDCl_3_, 400 MHz): δ 1.45 (9H, s, (CH_3_)_3_), 1.46 (9H, s, (CH_3_)_3_), 1.59–1.81 (4H, m, β-CH_2_, γ-CH_2_), 3.58–3.63 (2H, m, δ-CH_2_), 4.16–4.19 (1H, m, α-CH), 4.85 (2H, s, CH_2_), 5.08 (1H, d, *J* 8.0, NH), 7.38–7.40 (5H, m, 5 × Ar-CH), 8.21 (H, s, CHO); ^13^C NMR (CDCl_3_, 100 MHz): δ 171.6, 163.1, 155.4, 134.6, 129.4, 129.1, 128.8, 82.1, 79.7, 77.3, 53.5, 43.8, 30.1, 28.3, 28.0, 22.8; found (ESI^+^) 445.2332 [M + Na]^+^, C_22_H_34_N_2_O_6_Na requires 445.2309.

*tert-Butyl (S)-2-((((9H-fluoren-9-yl)methoxy)carbonyl)amino)-5-(N-(benzyloxy) formamido) pentanoate (***8***)*

A solution of (**6**) (3.5 g, 8.29 mmol) in 4 M HCl in dioxane (40 mL) was cooled to 0 °C. The reaction mixture was stirred at room temperature for 30 min. The solvent was removed under reduced pressure. The residue was dissolved in distilled DCM (50 mL) and treated with DIEA (4.18 mL, 24.0 mmol) for 15 min. Fmoc-Cl (2.48 g, 9.61 mmol) was added, and the reaction mixture was stirred at room temperature for 16 h. The solvent was evaporated and the residue was dissolved in sat. NaHCO_3_ (120 mL). The aqueous phase was washed with DCM (2 × 120 mL). The combined organic phase was concentrated, and the residue was subjected to chromatography (9:1 to 6:4 EtOAc: petroleum ether) to give (**8**) as an oil (3.05 g, 70%). Rf = 0.45 (1:1 EtOAc: petroleum ether); HPLC (System A, Gradient 1) R_t_ = 11.40 min; ^1^H NMR (CDCl_3_, 400 MHz): δ 1.37 (9H, s, (CH_3_)_3_), 1.59–1.81 (4H, m, β-CH_2_, γ-CH_2_), 3.23–3.53 (2H, m, δ-CH_2_), 4.14 (1H, t, *J* 6.9, CH), 4.18–4.20 (1H, m, α-CH), 4.30 (2H, d, *J* 6.9, CH_2_), 4.84 (2H, s, CH_2_), 5.33 (1H, s, NH), 7.21–7.33 (9H, m, 9 × Ar-CH), 7.51 (2H, d, *J* 7.3, 2 × Ar-CH), 7.67 (2H, d, *J* 7.5, 2 × Ar-CH), 8.14 (1H, s, CHO); ^13^C NMR (CDCl_3_, 100 MHz): δ 171.3, 163.2, 155.9, 143.9, 143.8, 141.3, 134.2, 129.6, 129.5, 129.2, 128.8, 128.5, 127.7, 127.1, 125.1, 120.0, 82.4, 77.8, 67.0, 54.0, 47.2, 43.6, 30.0, 28.0, 22.7; found (ESI^+^) 567.2473 [M + Na]^+^, C_28_H_28_N_2_O_6_Na requires 567.2466.

*(S)-2-((((9H-Fluoren-9-yl)methoxy)carbonyl)amino)-5-(N-(benzyloxy)formamido) pentanoic acid (***9***)* [17]

*Method A:* A solution of (**6**) (700 mg, 1.60 mmol) in DCM/TFA (1:1, *v*:*v*, 2 mL) was stirred at room temperature. After 3.5 h, the solvent was evaporated off. The residue was dissolved in H_2_O (4 mL) and NaHCO_3_ (536 mg, 6.40 mmol) was added. A solution of Fmoc-OSu (539 mg, 1.60 mmol) in THF (1 mL) was added to the reaction mixture, which was allowed to stir at room temperature for 23 h. The organic solvent was evaporated, and the aqueous solution was acidified to pH 2. The aqueous solution was then extracted with EtOAc (3 × 10 mL). The organic extract was dried over MgSO_4_, and the solvent was evaporated to give the crude product. The residue was subjected to chromatography (19:1:0.5 DCM:MeOH:AcOH) to give (**9**) (220 mg, 25%).

*Method B:* A solution of (**8**) (200 mg, 0.36 mmol) in 0.1 M HCl/HFIP (5 mL) was stirred at room temperature. After 4 h, the solvent was removed under reduced pressure to give (**9**) in quantitative yield.

*Data for* (**9**): Rf = 0.20 (1:9 MeOH:DCM); [α${]}_{D}^{22.0}$ = +7.3 (c 10.0, CHCl_3_) (lit. [17] [α${]}_{D}^{24.8}$ = +9.60 (c 10.0, CHCl_3_)); HPLC (System A, Gradient 1) R_t_ = 9.74 min; ^1^H NMR (CDCl_3_, 500 MHz): δ 1.62–1.92 (4H, m, β-CH_2_, γ-CH_2_), 3.61–3.69 (2H, m, δ-CH_2_), 4.23 (1H, t, *J* 6.6, α-CH), 4.38–4.45 (3H, m, CH, CH_2_), 4.83 (2H, s, CH_2_), 5.58 (1H, s, NH), 7.30–7.42 (9H, m, 9 × Ar-CH), 7.61 (2H, t, *J* 6.3, 2 × Ar-CH), 7.77 (2H, d, *J* 7.4, 2 × Ar-CH), 8.21 (1H, s, CHO); ^13^C NMR (CDCl_3_, 125 MHz): δ 175.0, 163.7, 156.2, 143.9, 143.7, 141.3, 134.0, 129.5, 129.3, 128.9, 128.6, 127.8, 127.1, 125.1, 120.0, 77.9, 67.1, 53.2, 47.1, 43.7, 29.5, 22.9; found (ESI^+^) 489.2027 [M + H]^+^, C_28_H_29_N_2_O_6_ requires 489.2020. The obtained data was consistent with that reported by Mashiach and Meijler [17].

### 3.3. Synthesis of Peptide Derivatives

*Fmoc-D-Ser(tBu)-Arg(Pbf)-D-Ser(tBu)-Orn(Boc)-Lys(Dde)-Orn(Boc)-Thr(tBu)-Thr(tBu)-2-chlorotrityl resin (***10***)*

2-Chlorotrityl resin (1.4 mmol/g, 0.50 g) was used for the solid-phase peptide synthesis. The resin was pre-swollen in an SPPS vessel using distilled DCM (5 mL). The first amino acid, Fmoc-Thr(tBu)-OH (0.77 g, 1.95 mmol), was loaded using DIEA (0.67 μL, 3.90 mmol) in DCM (5 mL) for 60 min. Fmoc deprotection was performed by incubating the resin with 20% piperidine in DMF (2 × 5 min) for 10 min. The resin loading was 1.2 mmol/g, confirmed by measuring the UV absorbance at 304 nm of the Fmoc deprotection solution [39]. For each coupling, amino acids were added in excess (3 eq), with DIEA (6 eq) and PyBOP (3 eq) for 90 min. The success of each coupling was confirmed by the Kaiser test [38]. For the coupling of Fmoc-Arg(Pbf)-OH, the above coupling conditions were repeated twice. HATU (2 eq) and TMP (4 eq) were used for the coupling of Fmoc-D-Ser(tBu)-OH. The peptide resin was finally washed thoroughly with DMF (3 × 5 mL), DCM (3 × 5 mL), MeOH (3 × 5 mL) and Et_2_O (3 × 5 mL), and dried in vacuo to give (**10**).

*Fmoc-D-Ser(tBu)-Arg(Pbf)-D-Ser(tBu)-Orn(OBn, CHO)-Lys(Mtt)-Orn(OBn, CHO)-Thr(tBu)-Thr(tBu)-2-chlorotrityl resin (***11***)*

The same procedure was followed as for peptide resin (**11**), except that HATU (2 eq) and DIEA (4 eq) were used for the coupling of the modified Orn derivative. The peptide resin was washed and dried as before to give (**11**).

*Fmoc-D-Ser(tBu)-Arg(Pbf)-D-Ser(tBu)-Orn(Boc)-Lys-Orn(Boc)-Thr(tBu)-Thr(tBu)-OH (***12***)*

Peptide resin (**10**) (50 mg) was swollen in an SPPS vessel with distilled DCM (5 mL) for 15 min and drained. Dde cleavage was achieved by treating the peptide on resin with a mixture of NH_2_OH · HCl/imidazole in NMP/DCM (5 mL) for 3 h [32]. Cleavage from the resin was achieved by incubating the resin with 1% TFA/DCM (0.5 mL), and the vessel was agitated for 2 min. The resin was filtered under reduced pressure, and the filtrate was collected in a flask containing pyridine/MeOH (1:9, *v*/*v*, 2 mL). The acid treatment step was repeated ten times, and the resin was washed with DCM (3 × 1.5 mL), MeOH (3 × 1.5 mL), DCM (3 × 1.5 mL) and MeOH (3 × 1.5 mL). The cleavage solution was concentrated to 5% of the original volume, and H_2_O (7 mL) was added. The white precipitate that was formed was collected by centrifugation. The crude material was washed with H_2_O (3 × 7 mL) and dried in vacuo to give linear peptide (**12**) (20 mg) as a white solid. Analytical HPLC (System A, Gradient 1) R_t_ = 10.2 min; found (ESI^+^) 903.5229 [M + 2H]^2+^, C_91_H_145_N_13_O_22_S requires 903.5362.

*Fmoc-D-Ser(tBu)-Arg(Pbf)-D-Ser(tBu)-Orn(OBn, CHO)-Lys-Orn(OBn, CHO)-Thr(tBu)-Thr(tBu)-OH (***13***)*

Peptide resin (**11**) (200 mg) was swollen in an SPPS vessel with distilled DCM (5 mL) for 5 min. Cleavage from the resin was achieved by incubating the resin with 1% TFA/DCM (1.5 mL), and the vessel was agitated for 2 min. The resin was filtered under reduced pressure, and the filtrate was collected in a flask containing pyridine/MeOH (1:9, *v*/*v*, 5 mL). The acid treatment step was repeated ten times, and the resin was washed with DCM (3 × 5 mL), MeOH (3 × 5 mL), DCM (3 × 5 mL) and MeOH (3 × 5 mL). The cleavage solution was concentrated to 5% of the original volume and H_2_O (20 mL) was added. The white precipitate was formed and collected by centrifugation. The crude material was washed with H_2_O (3 × 10 mL), dried in vacuo to give linear peptide (**13**) (70 mg) as a white solid; Analytical HPLC (System A, Gradient 1) R_t_ = 11.20 min; Analytical HPLC (System B, Gradient 1) R_t_ = 11.12 min; found (ESI^+^) 937.5072 [M + 2H]^2+^, C_96_H_140_N_14_O_22_S requires 937.5066.

*Fmoc-D-Ser(tBu)-Arg(Pbf)-D-Ser(tBu)-Orn(Boc)-cyclo-(-Lys-Orn(Boc)-Thr(tBu)-Thr(tBu)-) (***14***)*

*Method A (10 eq HATU):* A solution of (**12**) (5 mg, 2.77 µmol), HATU (10.5 mg, 27.7 µmol), and DIEA (4.8 µL, 27.7 µmol) in anhydrous DMF (5 mL) was stirred at room temperature. The reaction progress was monitored by analytical HPLC (System A, Gradient 1), which showed the complete disappearance of starting material at 10.54 min and the generation of new species at 12.62, 12.89, and 13.10 min after 2 h (Supplementary Material, Figure S2). The desired compound could be detected by mass spectrometry.

*Method B (10 eq PyBOP):* A solution of (**12**) (5 mg, 2.77 µmol), PyBOP (14.4 mg, 27.7 µmol), and DIEA (4.8 µL, 27.7 µmol) in anhydrous DMF (5 mL) was stirred at room temperature. After 1 h, the disappearance of starting material at 10.54 min and the appearance of a new species at 13.10 min were observed. The solvent was removed under reduced pressure, and the residue was purified by preparative HPLC (Gradient 2) to give (**14**) (2 mg, 40%).

*Data for (***14***):* HPLC (System A, Gradient 1) R_t_ = 13.10 min; found (ESI^+^) 895.0188 [M + 2H]^2+^, C_90_H_142_N_14_O_21_S requires 895.0190.

*Fmoc-D-Ser(tBu)-Arg(Pbf)-D-Ser(tBu)-Orn(OBn, CHO)-cyclo-(-Lys-Orn(OBn, CHO)-Thr(tBu)-Thr(tBu)-) (***15***)*

*Method A (10 eq HATU):* A solution of (**13**) (5 mg, 2.66 µmol), HATU (10.1 mg, 26.6 µmol), and DIEA (4.6 µL, 26.6 µmol) in anhydrous DMF (5 mL) was stirred at room temperature. The reaction progress was monitored by analytical HPLC (System B, Gradient 1). After 48 h, incomplete consumption of starting material at 11.20 min and the generation of multiple species were observed (Supplementary Material, Figure S1). The solvent was removed under reduced pressure, and the residue was purified by preparative HPLC (Gradient 2). The desired compound could be detected by mass spectrometry.

*Method B (10 eq PyBOP):* A solution of (**13**) (5 mg, 2.66 µmol), PyBOP (13.9 mg, 26.6 µmol), and DIEA (4.6 µL, 26.62 µmol) in anhydrous DMF (5 mL) was stirred at room temperature. The reaction progress was monitored by analytical HPLC (System B, Gradient 1), which showed the generation of multiple peaks around the starting material at 11.20 min after 10 h.

*Method C (1.2 eq PyBOP):* A solution of (**13**) (5.0 mg, 2.7 µmol), PyBOP (1.7 mg, 3.2 µmol), and DIEA (0.9 µL, 5.32 µmol) in anhydrous DMF (5 mL) was stirred at room temperature. The reaction progress was monitored by analytical HPLC (System B, Gradient 1). After 14 h, partial consumption of starting material at 11.20 min and the generation of a new species at 12.74 min were observed. The solvent was removed under reduced pressure, and the residue was purified by preparative HPLC (Gradient 2) to give (**15**) (2 mg, 41%).

*Data for (***15***):* HPLC (System B, Gradient 1) R_t_ = 12.74 min; found (ESI^+^) 929.0027 [M + 2H]^2+^, C_96_H_138_N_14_O_21_S requires 929.0029.

*Fmoc-D-Ser-Arg(Pbf)-D-Ser-Orn(OBn, CHO)-Lys-Orn(OBn, CHO)-Thr-Thr-OH (***16***) and Fmoc-D-Ser-Arg-D-Ser-Orn(OBn, CHO)-Lys-Orn(OBn, CHO)-Thr-Thr-OH (***17***)*

*Method A:* A solution of (**13**) (15 mg, 8.01 µmol) in 0.1 M HCl/HFIP (2 mL) was stirred at room temperature. The reaction progress was monitored by analytical HPLC (System A, Gradient 1), which showed the disappearance of starting material at 11.20 min and the generation of a new species at 8.30 min after 10 min. After stirring for 30 min, the peak at 8.30 min had reduced and the formation of another new species at 6.70 min was observed (Supplementary Material Figure S4). The solvent was evaporated, and the residue was purified by preparative HPLC (Gradient 2) to give (**16**) (3 mg, 23%) and (**17**) (4 mg, 35%).

*Method B:* A solution of (**13**) (2 mg, 1.06 µmol) in 0.1 M HCl/HFIP (0.5 mL) was stirred at room temperature. The reaction was monitored by HPLC analysis (System A, Gradient 1), which showed the disappearance of starting material at 11.20 min and the appearance of a new species at 6.70 min after 1 h. The solvent was removed under reduced pressure, and the residue was purified by preparative HPLC (Gradient 2) to give (**17**) (0.8 mg, 54%).

*Method C:* A solution of (**13**) (80 mg, 42.7 µmol) in 0.1 M HCl/HFIP (5 mL) was stirred at room temperature. After 10 min, the solvent was removed under reduced pressure, and the residue was purified by preparative HPLC (Gradient 2) to give (**16**) (66 mg, 92%).

*Data for (***16***):* HPLC (System A, Gradient 1) R_t_ = 8.30 min; found (ESI^+^) 828.3817 [M + 2H]^2+^, C_80_H_108_N_14_O_22_S requires 825.3820.

*Data for (***17***):* HPLC (System A, Gradient 1) R_t_ = 6.70 min; found (ESI^+^) 699.3412 [M + 2H]^2+^, C_67_H_92_N_14_O_19_ requires 699.3404.

*Fmoc-D-Ser-Arg(Pbf)-D-Ser-Orn(OBn, CHO)-cyclo-(-Lys-Orn(OBn, CHO)-Thr-Thr-) (***18***)*

*Method A:* A solution of (**16**) (66 mg, 40.05 µmol), PyBOP (62.5 mg, 120.2 µmol) and DIEA (31.06 mg, 240.30 µmol) in anhydrous DMF (50 mL) was stirred at room temperature. The reaction progress was monitored by HPLC (System B, Gradient 1), which showed the complete disappearance of starting material at 8.30 min and the generation of a new species at 9.08 min. The solvent was removed under reduced pressure, and the residue was purified by preparative HPLC (Gradient 2) to give (**18**) (25 mg, 40%).

*Method B:* A solution of linear peptide (**13**) (20 mg, 10.70 µmol) in 0.1 M HCl/HFIP (5 mL) was stirred at room temperature and the reaction was monitored by analytical HPLC (System B, Gradient 1). The complete disappearance of starting linear peptide at 11.12 min and the formation of a new species at 8.30 min were observed after 10 min. The solvent was removed under reduced pressure, and the residue was dissolved in anhydrous DMF (20 mL). The resulting solution was immediately neutralized with DIEA (8.30 mg, 64.2 µmol). PyBOP (16.7 mg, 32.2 µmol) was then added, and the reaction mixture was stirred at room temperature. After 1 h, the disappearance of deprotected intermediate at 8.30 min and the formation of a new species at 9.08 min were observed by HPLC analysis. The solvent was evaporated, and the residue was purified by preparative HPLC (Gradient 2) to give (**18**) (12 mg, 70%).

*Data for (***18***):* HPLC (System A, Gradient 1) R_t_ = 9.09 min; HPLC (System B, Gradient 1) R_t_ = 9.08 min; found (ESI^+^) 816.3780 [M + 2H]^2+^, C_80_H_106_N_14_O_21_S requires 816.3762.

## Data Availability

Data is contained within the article or the Supplementary Materials.

## Supplementary Materials

The following supporting information can be downloaded at https://www.mdpi.com/article/10.3390/molecules31121988/s1, Figure S1. HPLC chromatograms for the cyclization of 13. Figure S2. HPLC chromatograms for the cyclization of 12. Figure S3. HPLC chromatograms for the cyclization of 13. Figure S4. HPLC chromatograms for the treatment of 13 with 0.1M HCl in HFIP. Figure S5. HPLC chromatograms for the cyclization of 16.
