# Supplementary material for: Towards the Synthesis of Pyoverdines: Preparation and Reactivity of the N-Formylhydroxyornithine Residue
Source: Molecules. 2026 Jun 6;31(12):1988. doi: 10.3390/molecules31121988 (PMC13305306; doi:10.3390/molecules31121988)
Supplement: Supplementary file 1 [file molecules-31-01988-s001.zip › molecules-4272760-supplementary.pdf]

Supplementary Material

Towards the synthesis of pyoverdines: preparation and reactivity of the *N*-formylhydroxyornithine residue

Tianzhu Zhang,[1] Albert Bolhuis[1] and Ian M. Eggleston[1*]

[1] Department of Life Sciences
University of Bath
Bath BA2 7AY, UK
E-mail: ie203@bath.ac.uk

Contents

Stability test for compound (8) under Dde cleavage conditions **2**

NMR characterisation data **3**

HPLC chromatograms of purified peptides **9**

HPLC chromatograms for cyclisations of peptides (12) and (13) and selective deprotection of peptide (13) .... Error! Bookmark not defined.

Reference **20**

Stability test for compound (8) under Dde cleavage conditions [34]

A suspension of $NH_2OH.HCl$ (1.25 g, 1.80 mmol), imidazole (0.92 g, 1.35 mmol) in NMP (5 mL) was sonicated at 30°C until dissolution was complete. This solution was diluted with DCM (5 mL) and used immediately. Compound **8** (2.0 mg, 3.7 μmol) was treated with a portion of the previously prepared solution (2 mL) and the reaction mixture was stirred at room temperature. The reaction progress was monitored by analytical HPLC (System A, Gradient 1) which showed the disappearence of starting material at 11.40 min and the generation of a new species at 8.72 min after 30 min. Mass spectrometry confirmed that the loss of the formyl group from **8** had occurred (found (ESI+) 517.2697 $[M+Na]^+$, $C_{31}H_{36}N_2O_5Na$ requires 517.2697).

NMR characterisation data

(2)

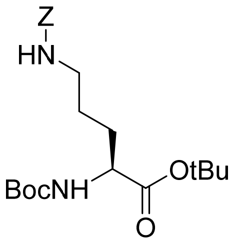

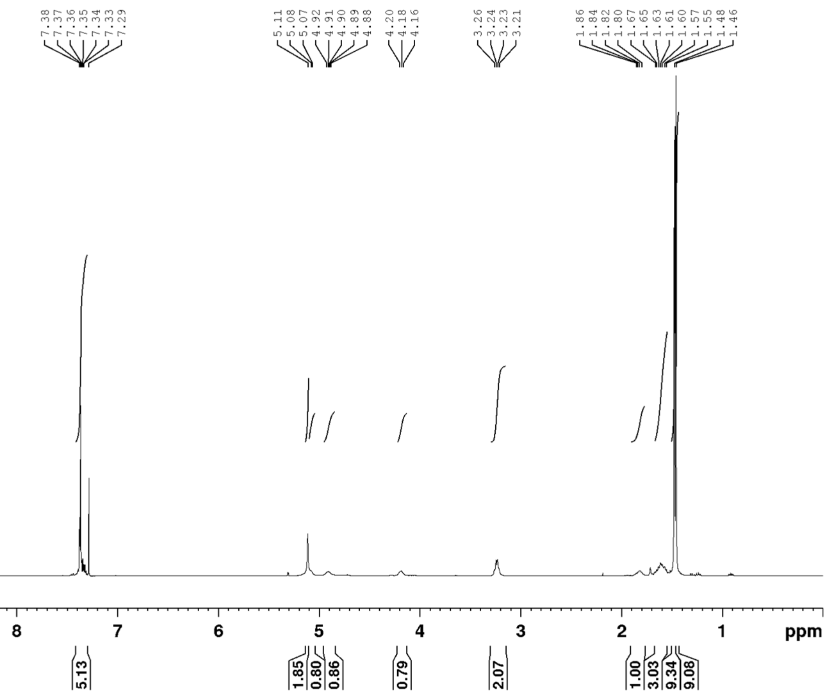

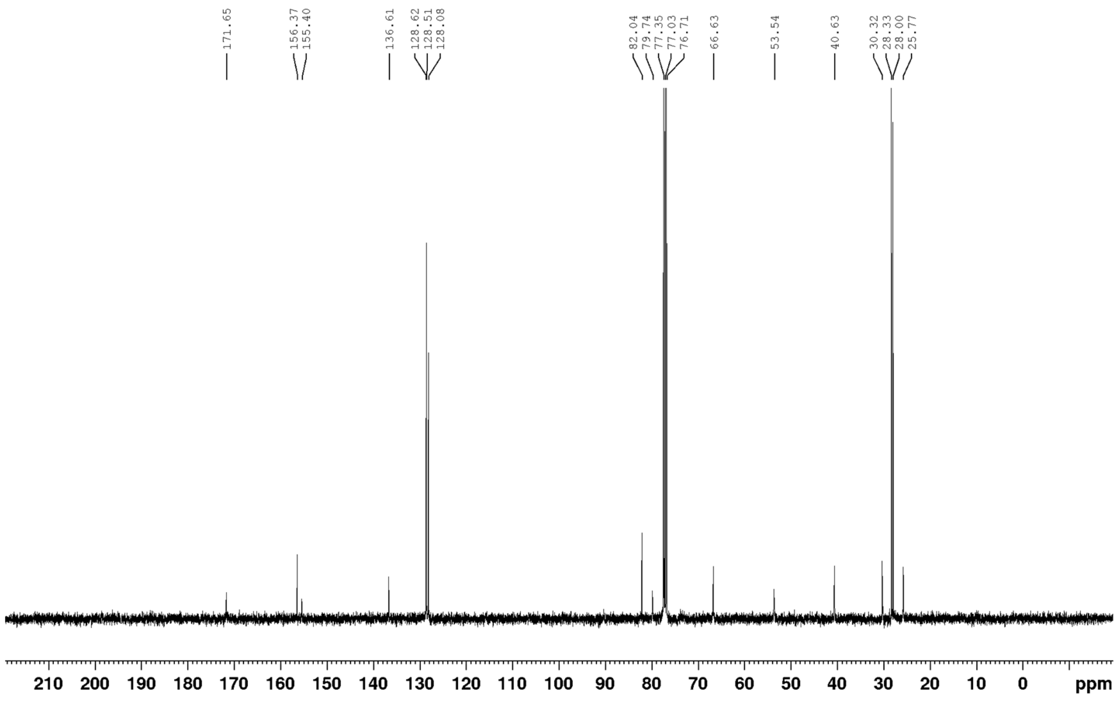

(3)

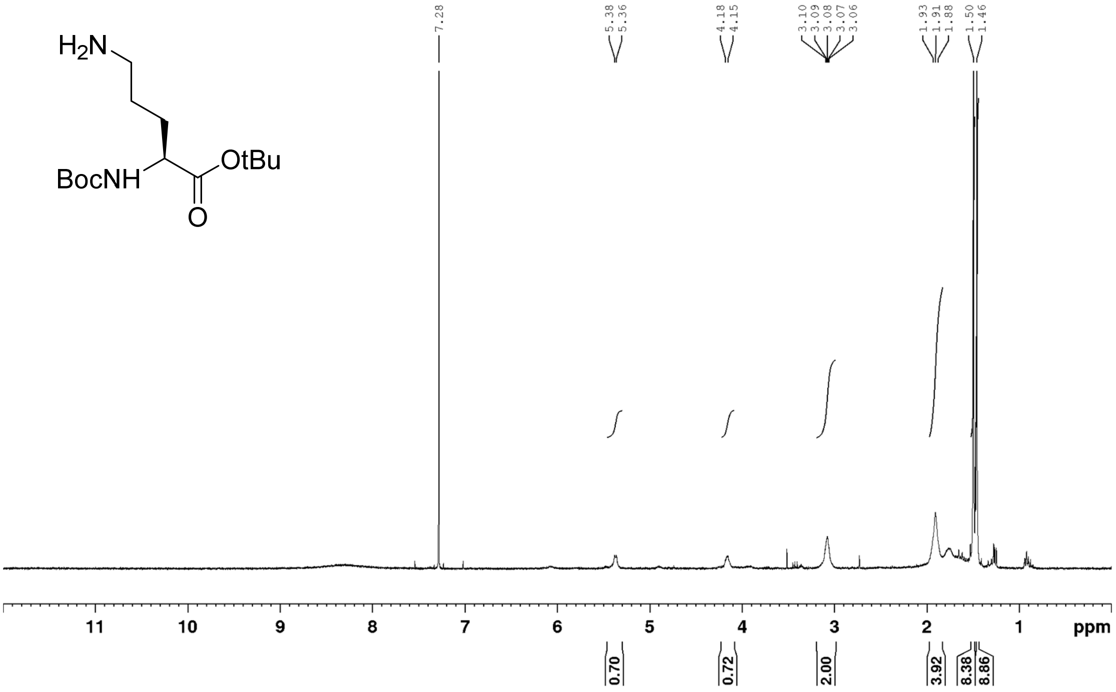
H2N
OtBu
BocNH
O
7.28
5.38
5.36
4.18
4.15
3.10
3.09
3.08
3.07
3.06
1.93
1.91
1.88
1.50
1.46
11 10 9 8 7 6 5 4 3 2 1 ppm
0.70
0.72
2.00
3.92
8.38
8.86

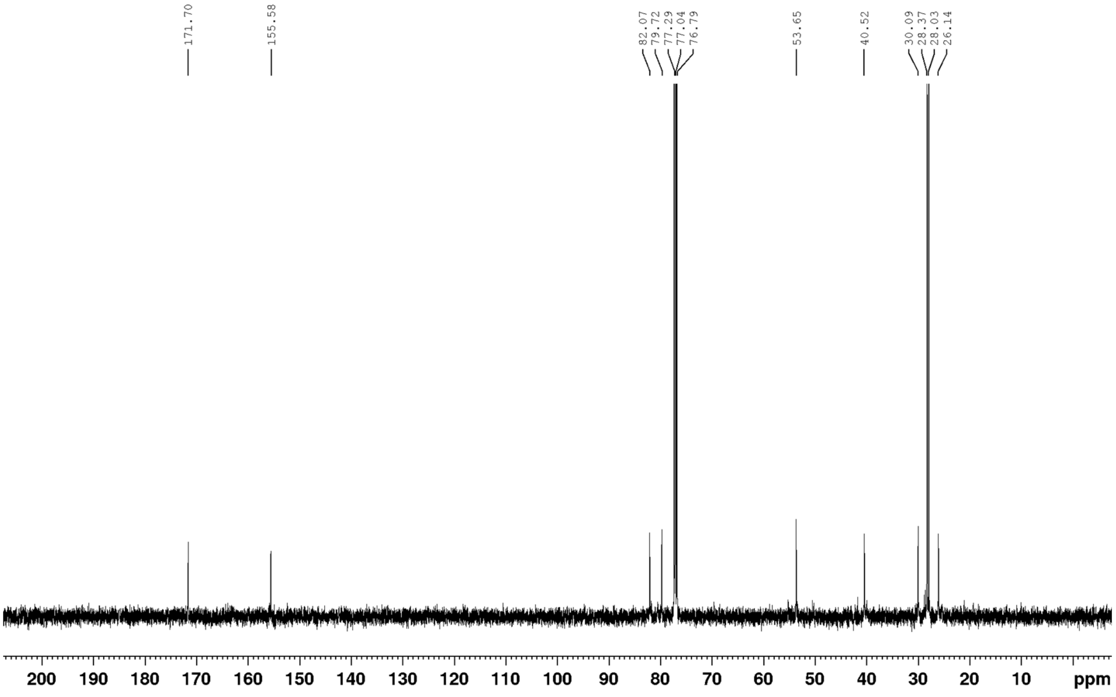
171.70
155.58
82.07
79.72
77.29
77.04
76.79
53.65
40.52
30.09
28.37
28.03
26.14
200 190 180 170 160 150 140 130 120 110 100 90 80 70 60 50 40 30 20 10 ppm

(4)

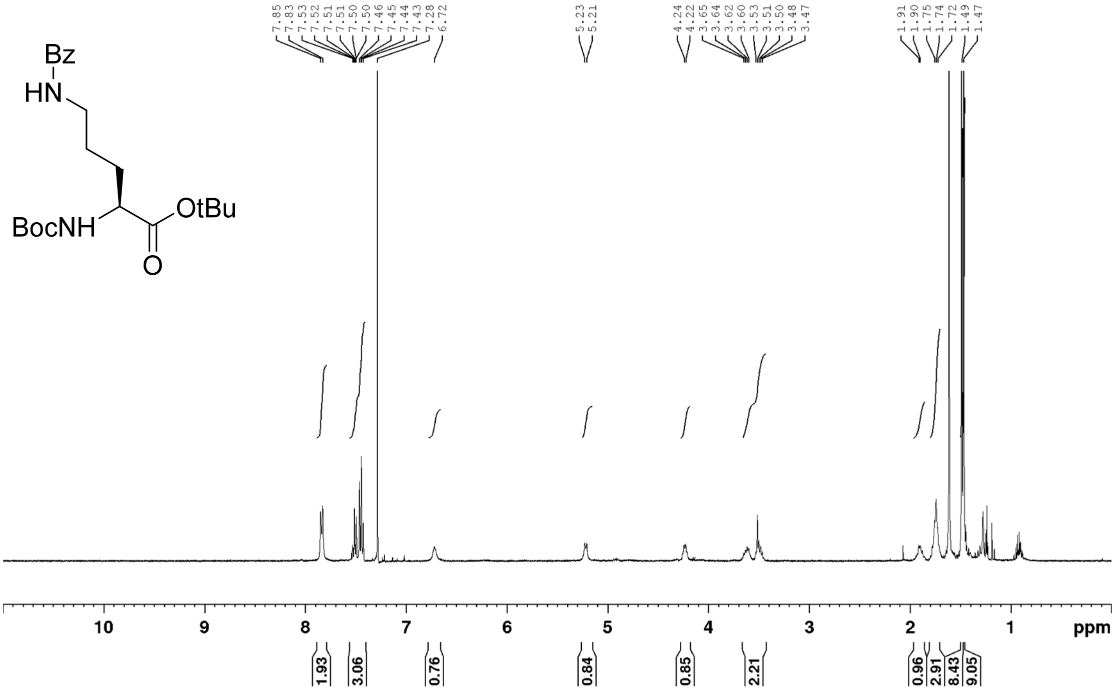
Bz
HN
BocNH
OtBu
O
7.85 7.83 7.53 7.52 7.51 7.51 7.50 7.50 7.46 7.45 7.44 7.43 7.28 6.72
5.23 5.21
4.24 4.22 3.65 3.64 3.62 3.60 3.53 3.51 3.50 3.48 3.47
1.91 1.90 1.75 1.74 1.72 1.49 1.47
10 9 8 7 6 5 4 3 2 1 ppm
1.93 3.06 0.76 0.84 0.85 2.21 0.96 2.91 8.43 9.05

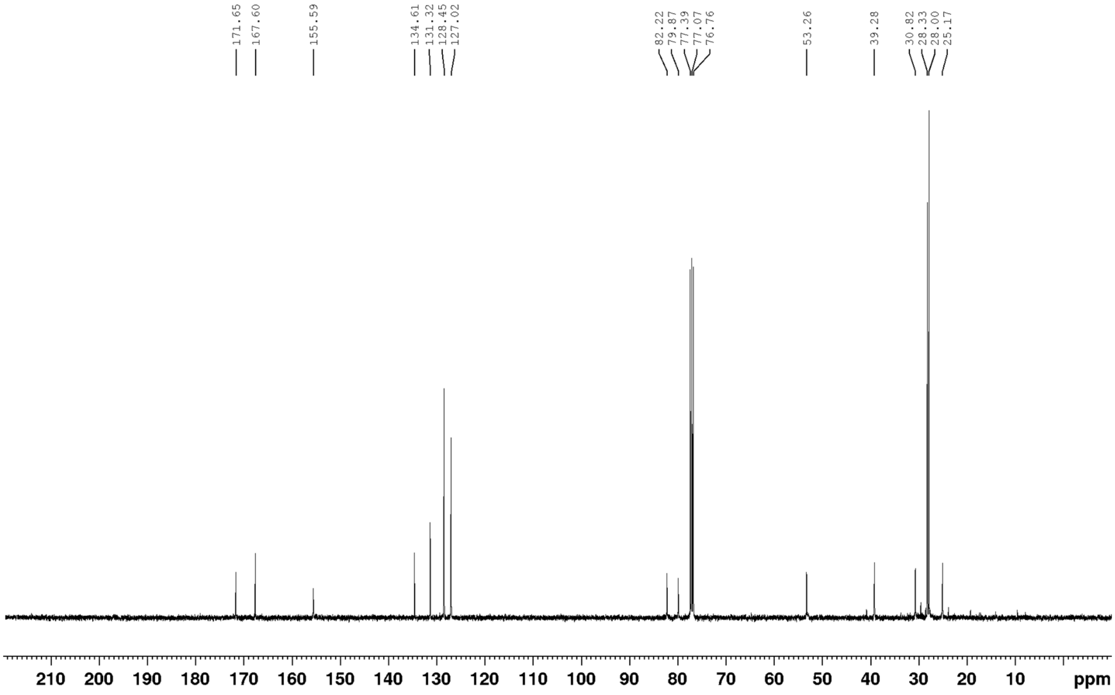
171.65 167.60 155.59 134.61 131.32 128.45 127.02
82.22 79.87 77.39 77.07 76.76 53.26 39.28 30.82 28.33 28.00 25.17
210 200 190 180 170 160 150 140 130 120 110 100 90 80 70 60 50 40 30 20 10 ppm

(5)

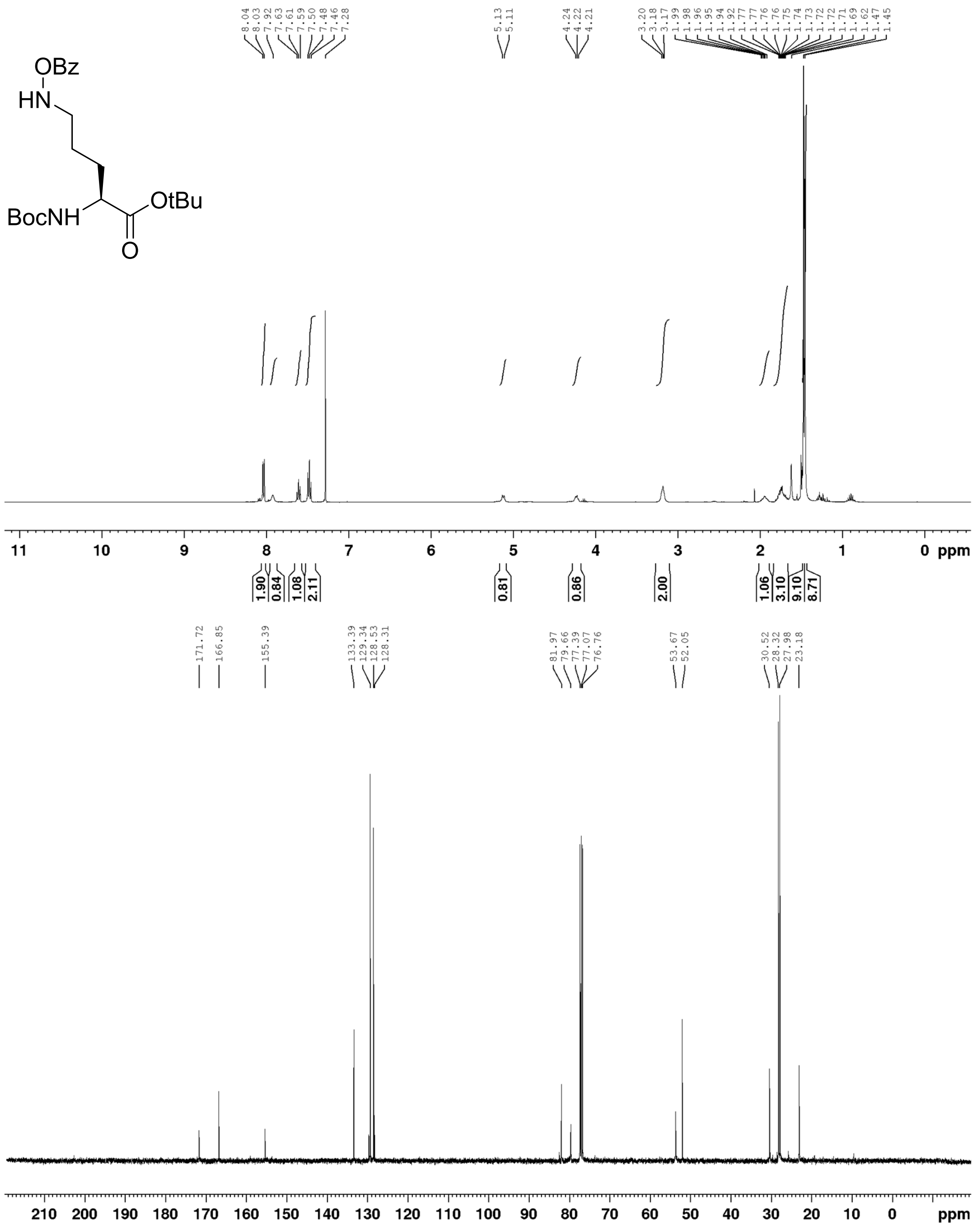
OBz
HN
BocNH
OtBu
O
8.04 8.03 7.92 7.63 7.61 7.59 7.50 7.48 7.46 7.28
5.13 5.11
4.24 4.22 4.21
3.20 3.18 3.17 1.99 1.98 1.96 1.95 1.94 1.92 1.77 1.77 1.76 1.76 1.75 1.74 1.73 1.73 1.72 1.72 1.71 1.69 1.62 1.47 1.45
11 10 9 8 7 6 5 4 3 2 1 0 ppm
1.90 0.84 1.08 2.11 0.81 0.86 2.00 1.06 3.10 9.10 8.71
171.72 166.85 155.39 133.39 129.34 128.53 128.31 81.97 79.66 77.39 77.07 76.76 53.67 52.05 30.52 28.32 27.98 23.18
210 200 190 180 170 160 150 140 130 120 110 100 90 80 70 60 50 40 30 20 10 0 ppm

(6)

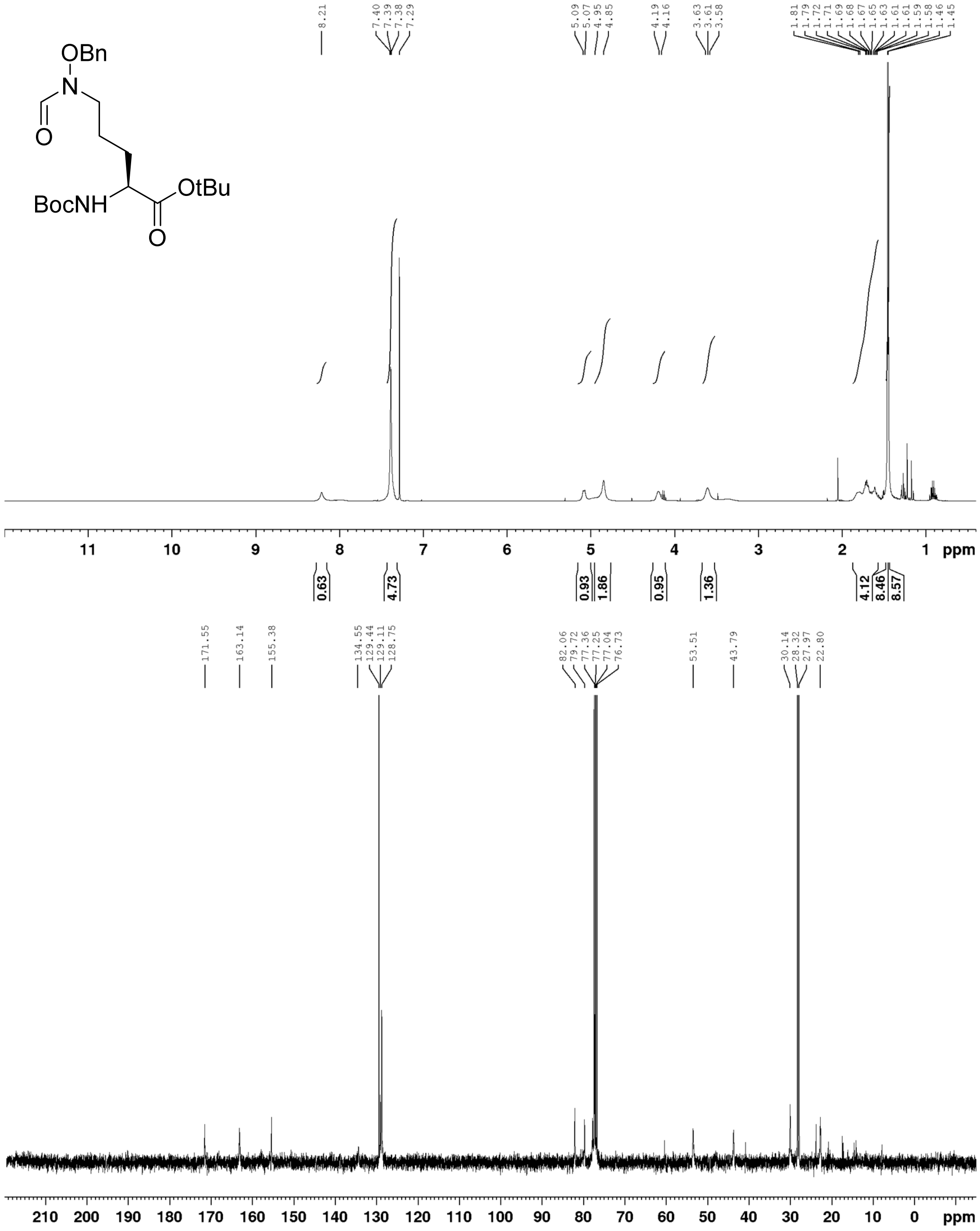
OBn
N
O
BocNH
OtBu
O
8.21
7.40 7.39 7.38 7.29
5.09 5.07 4.95 4.85
4.19 4.16
3.63 3.61 3.58
1.81 1.79 1.72 1.71 1.69 1.68 1.67 1.65 1.63 1.61 1.61 1.59 1.58 1.46 1.45
11 10 9 8 7 6 5 4 3 2 1 ppm
0.63 4.73 0.93 1.86 0.95 1.36 4.12 8.46 8.57
171.55 163.14 155.38
134.55 129.44 129.11 128.75
82.06 79.72 77.36 77.25 77.04 76.73
53.51 43.79
30.14 28.32 27.97 22.80
210 200 190 180 170 160 150 140 130 120 110 100 90 80 70 60 50 40 30 20 10 0 ppm

(8)

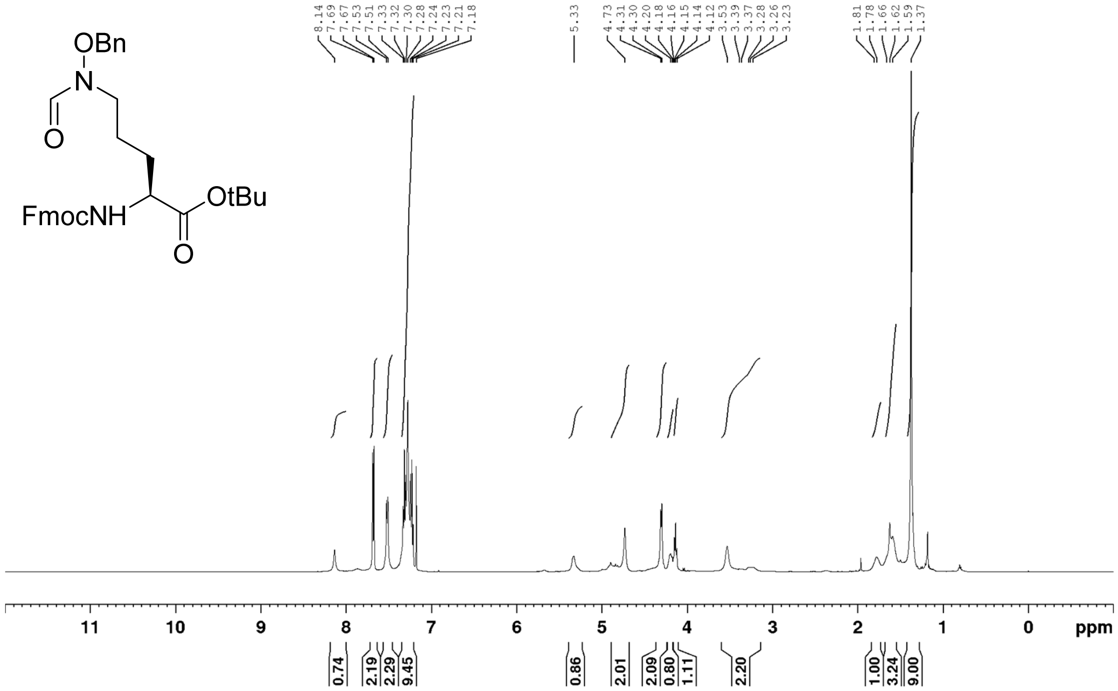
OBn
N
O
FmocNH
OtBu
O
8.14
7.69
7.67
7.53
7.51
7.33
7.32
7.30
7.28
7.24
7.23
7.21
7.18
5.33
4.73
4.31
4.30
4.20
4.18
4.16
4.15
4.14
4.12
3.53
3.39
3.37
3.28
3.26
3.23
1.81
1.78
1.66
1.62
1.59
1.37
11 10 9 8 7 6 5 4 3 2 1 0 ppm
0.74
2.19
2.29
9.45
0.86
2.01
2.09
0.80
1.11
2.20
1.00
3.24
9.00

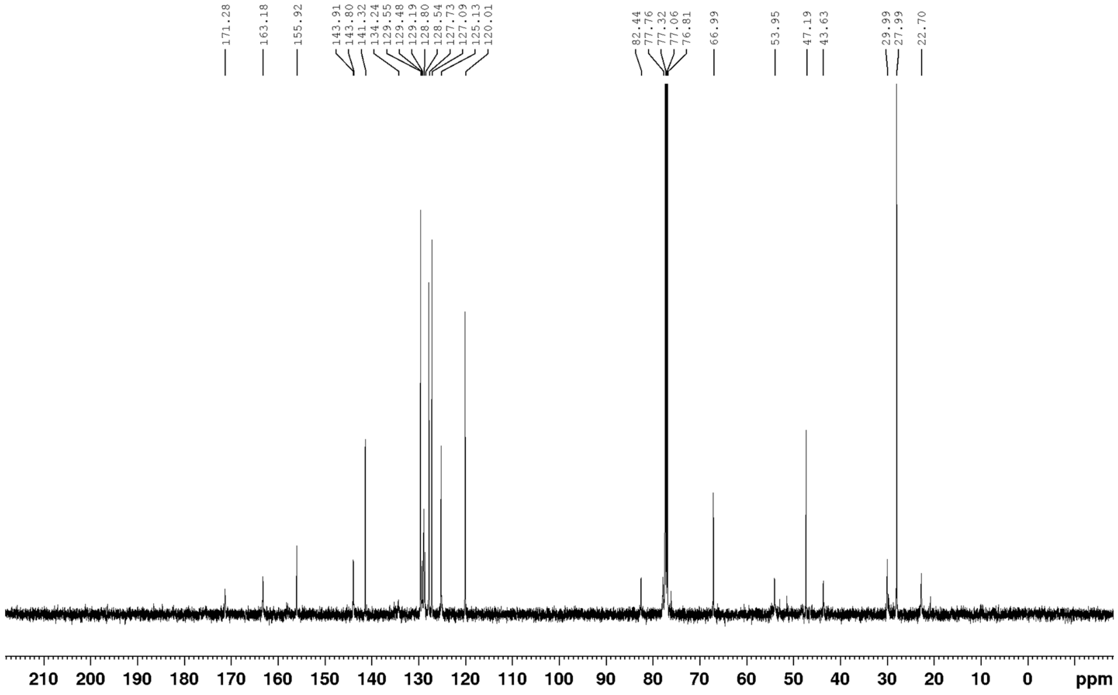
171.28
163.18
155.92
143.91
143.80
141.32
134.24
129.55
129.48
129.19
128.80
128.54
127.73
127.09
125.13
120.01
82.44
77.76
77.32
77.06
76.81
66.99
53.95
47.19
43.63
29.99
27.99
22.70
210 200 190 180 170 160 150 140 130 120 110 100 90 80 70 60 50 40 30 20 10 0 ppm

(9)

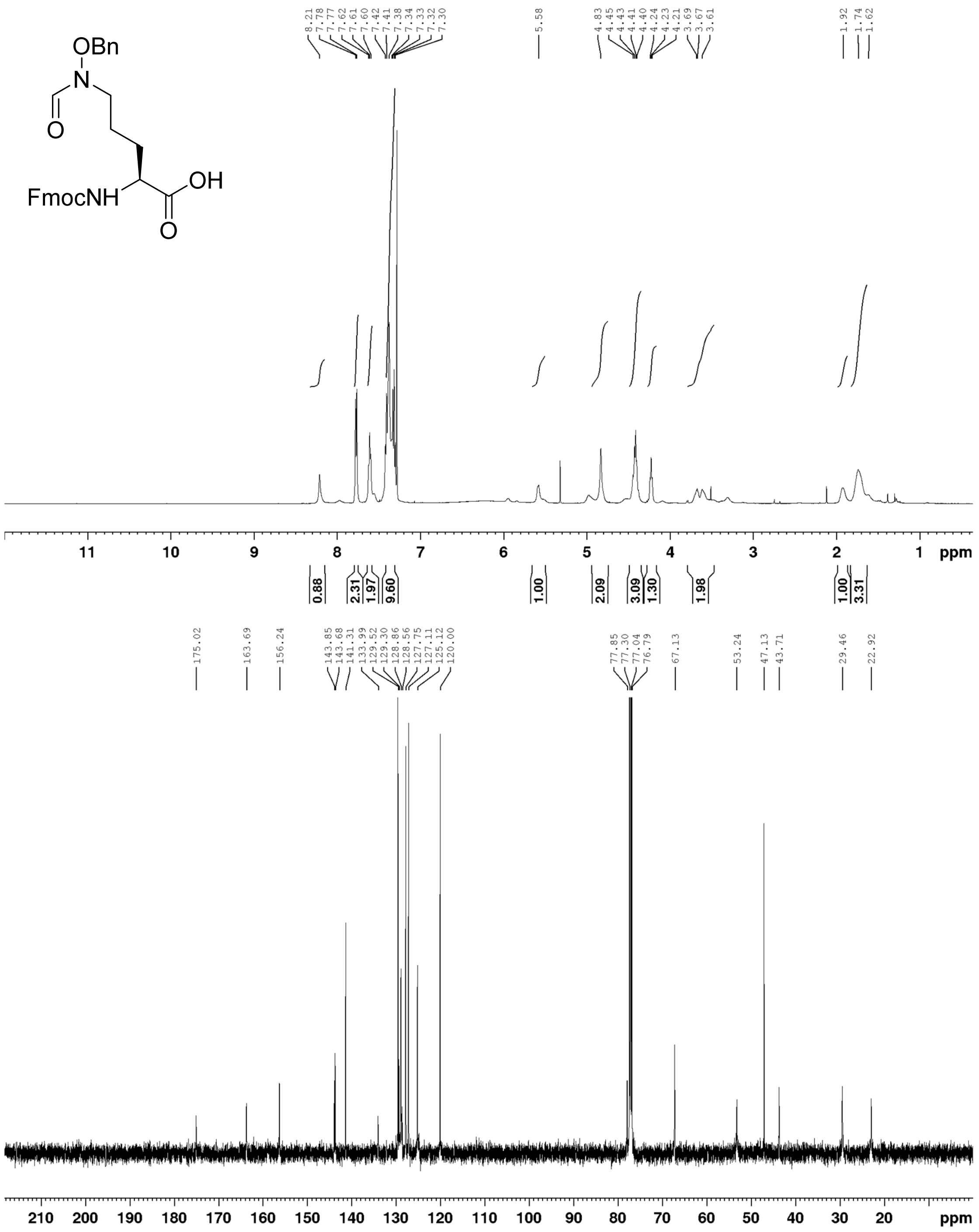
OBn
N
O
FmocNH
OH
O
8.21 7.78 7.77 7.62 7.61 7.60 7.42 7.41 7.38 7.34 7.33 7.32 7.30
5.58
4.83 4.45 4.43 4.41 4.40 4.24 4.23 4.21 3.69 3.67 3.61
1.92 1.74 1.62
11 10 9 8 7 6 5 4 3 2 1 ppm
0.88 2.31 1.97 9.60 1.00 2.09 3.09 1.30 1.98 1.00 3.31
175.02
163.69
156.24
143.85 143.68 141.31 133.99 129.52 129.30 128.86 128.56 127.75 127.11 125.12 120.00
77.85 77.30 77.04 76.79
67.13
53.24
47.13
43.71
29.46
22.92
210 200 190 180 170 160 150 140 130 120 110 100 90 80 70 60 50 40 30 20 ppm

HPLC chromatograms of purified peptides

(12)

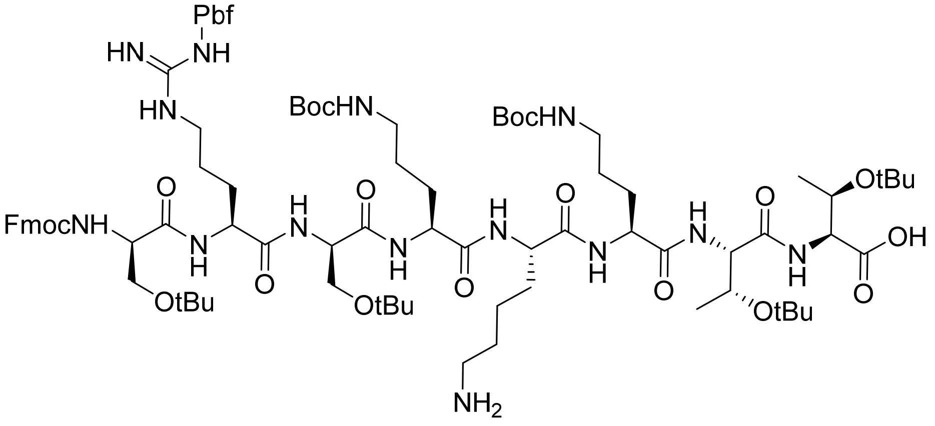

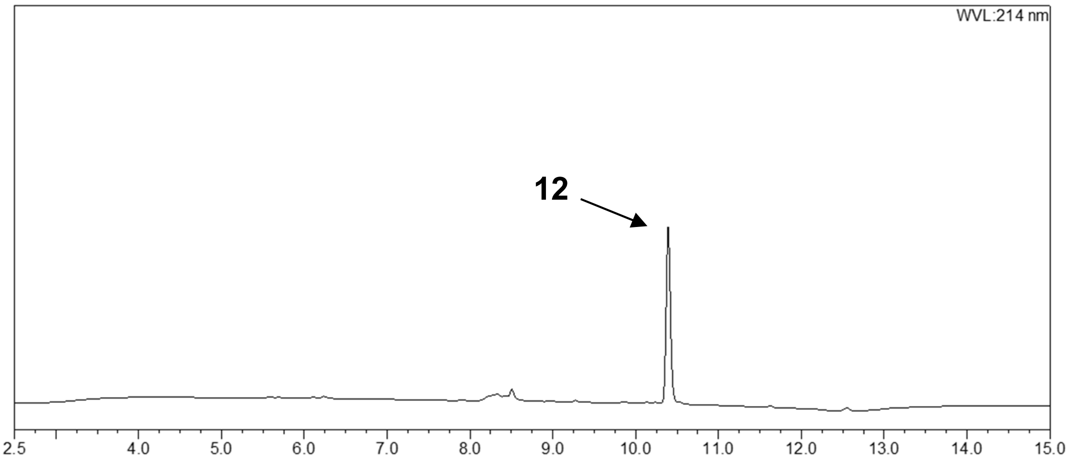

(13)

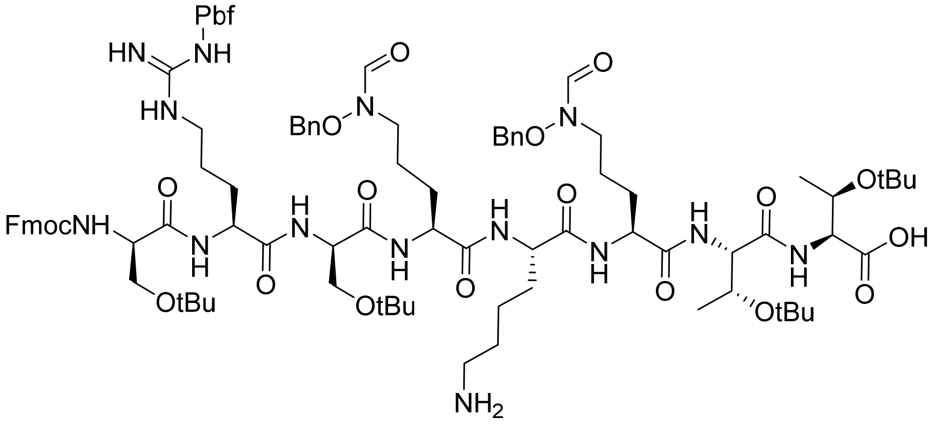

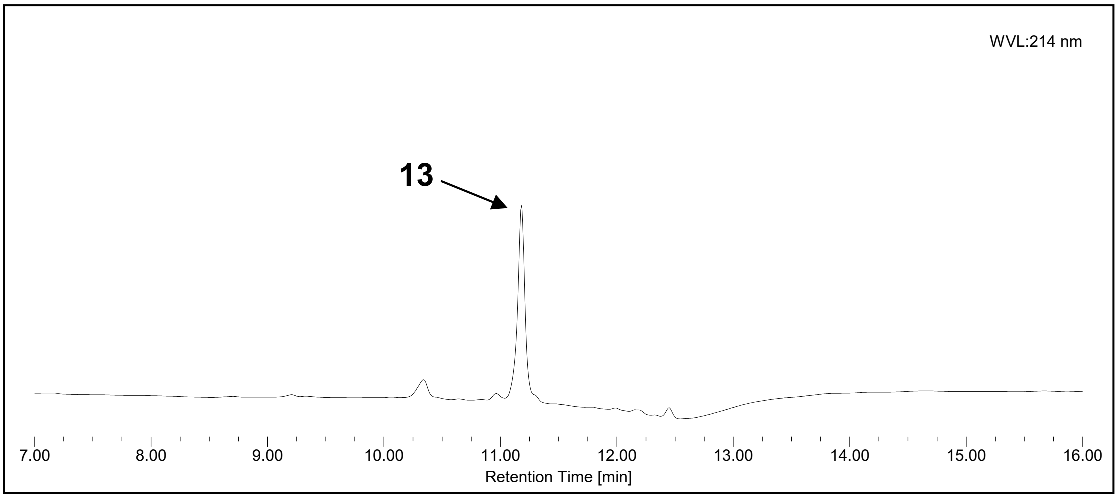
WVL:214 nm
13
7.00
8.00
9.00
10.00
11.00
12.00
13.00
14.00
15.00
16.00
Retention Time [min]

(14)

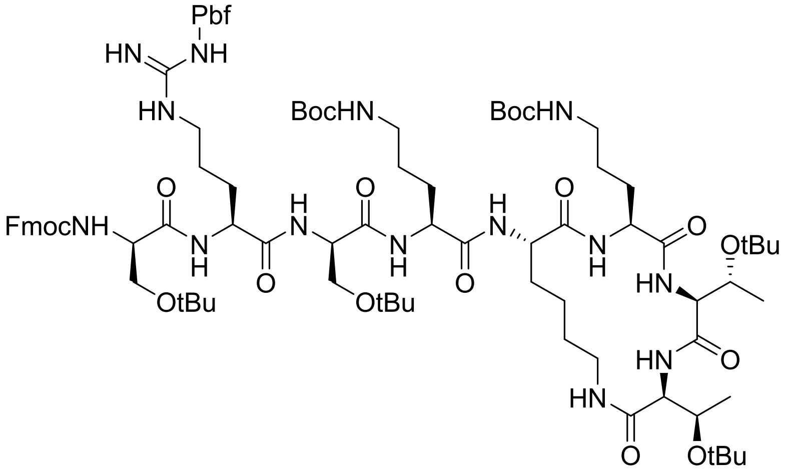
Pbf
HN
NH
HN
BocHN
BocHN
FmocNH
O
N
H
O
N
H
O
N
H
O
N
H
O
H
N
O
N
H
O
H
N
O
OtBu
OtBu
OtBu
HN
HN
O
HN
O
OtBu

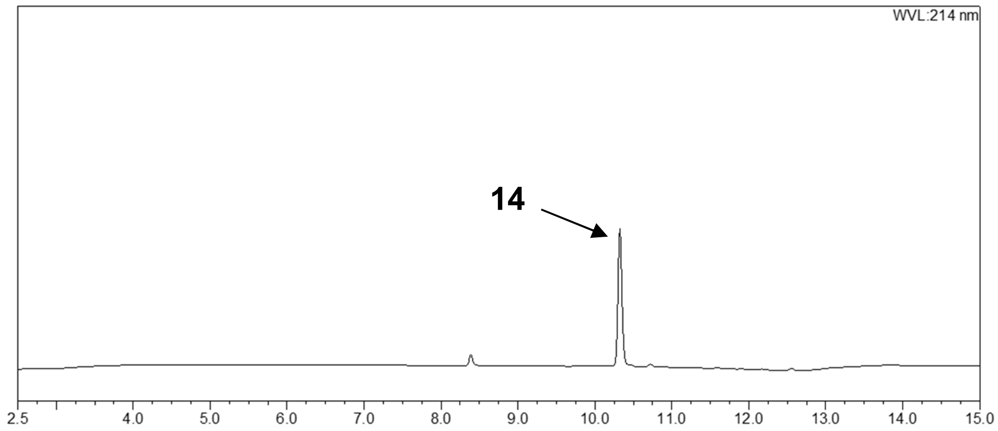
WVL:214 nm
14
2.5
4.0
5.0
6.0
7.0
8.0
9.0
10.0
11.0
12.0
13.0
14.0
15.0

(15)

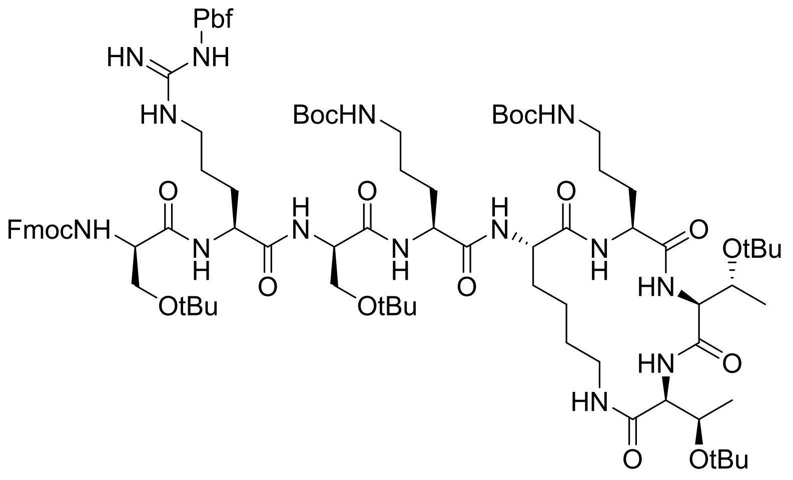
Pbf
HN
NH
HN
BocHN
BocHN
FmocNH
OtBu
OtBu
OtBu
OtBu
HN
HN
HN

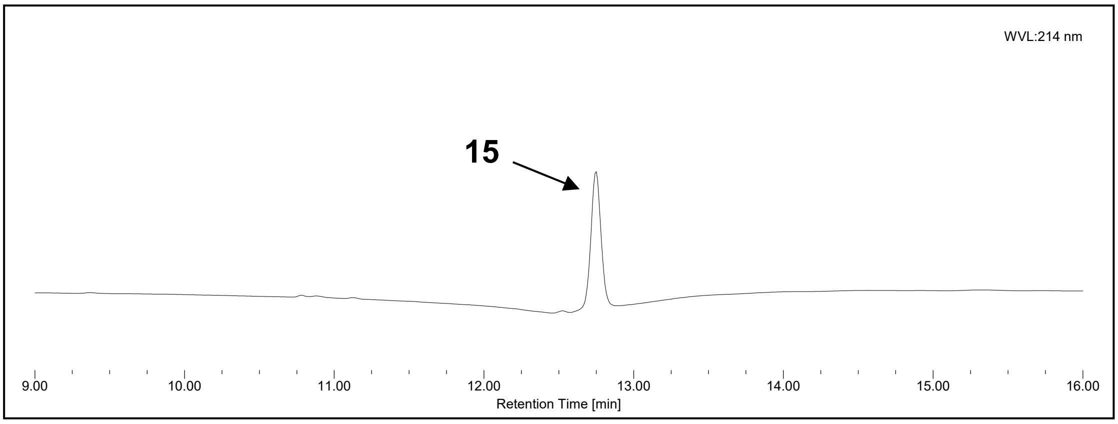
WVL:214 nm
15
9.00
10.00
11.00
12.00
13.00
14.00
15.00
16.00
Retention Time [min]

(16)

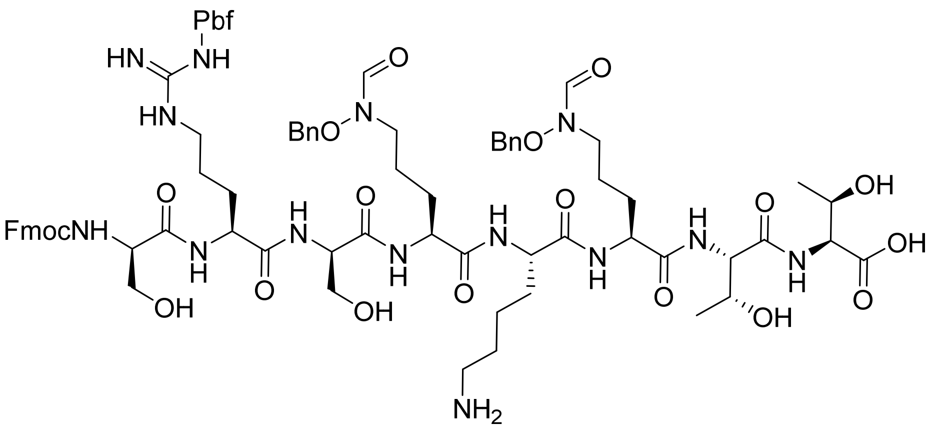
Pbf
HN
NH
HN
BnO
N
O
FmocNH
OH
OH
NH2
OH
OH
OH

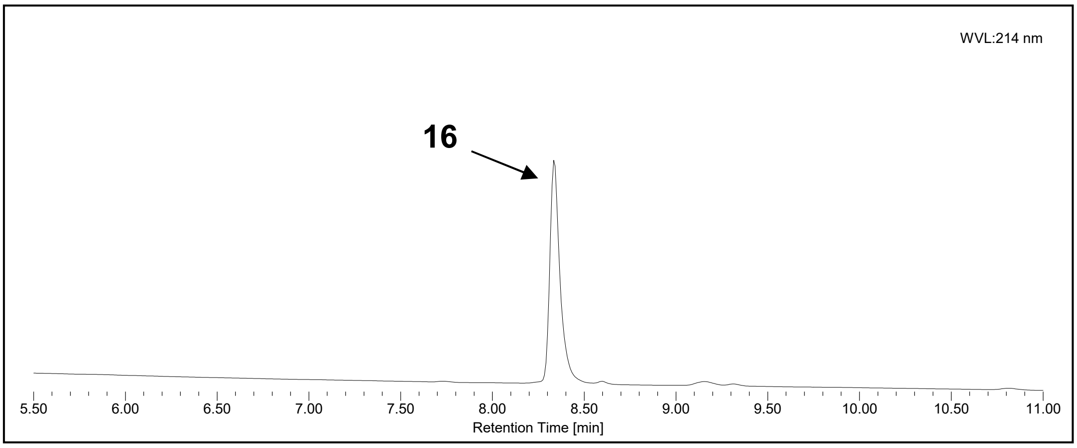
WVL:214 nm
16
5.50
6.00
6.50
7.00
7.50
8.00
8.50
9.00
9.50
10.00
10.50
11.00
Retention Time [min]

(17)

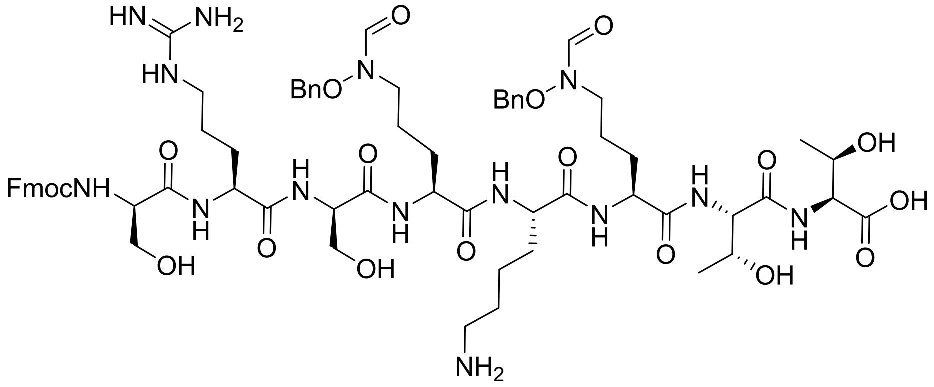

HN
NH2
HN
O
BnO
N
O
BnO
N
FmocNH
O
N
H
H
N
O
N
H
H
N
O
N
H
H
N
O
N
H
OH
OH
O
O
O
O
OH
OH
NH2

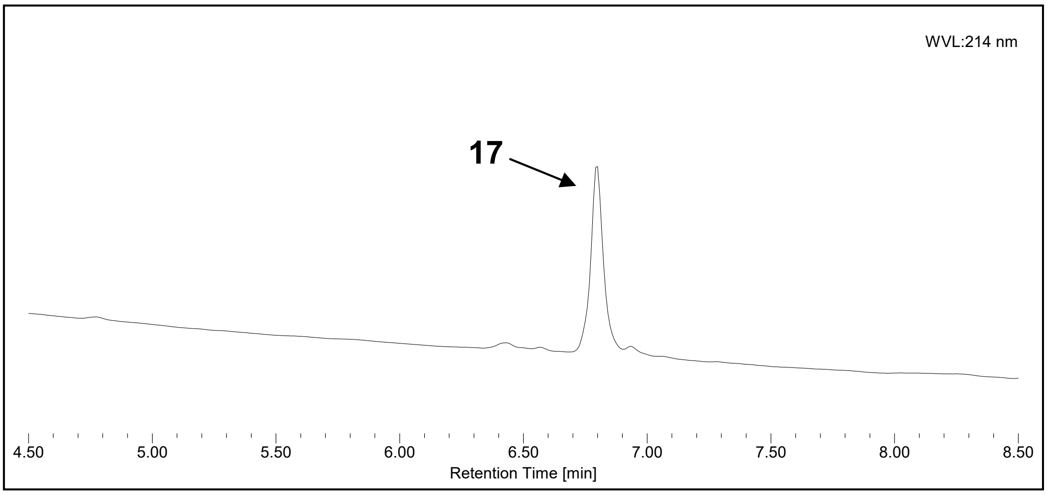

WVL:214 nm
17
4.50
5.00
5.50
6.00
6.50
7.00
7.50
8.00
8.50
Retention Time [min]

(18)

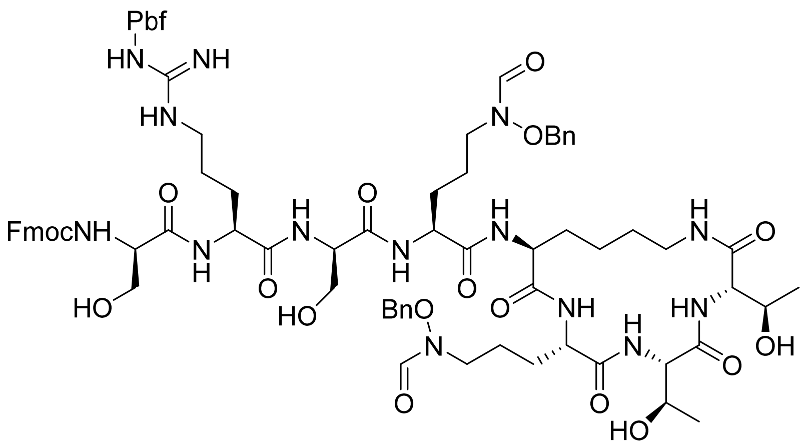
Pbf
HN
NH
HN
FmocNH
HO
H
N
O
HO
N
H
OBn
BnO
NH
HN
OH
HO

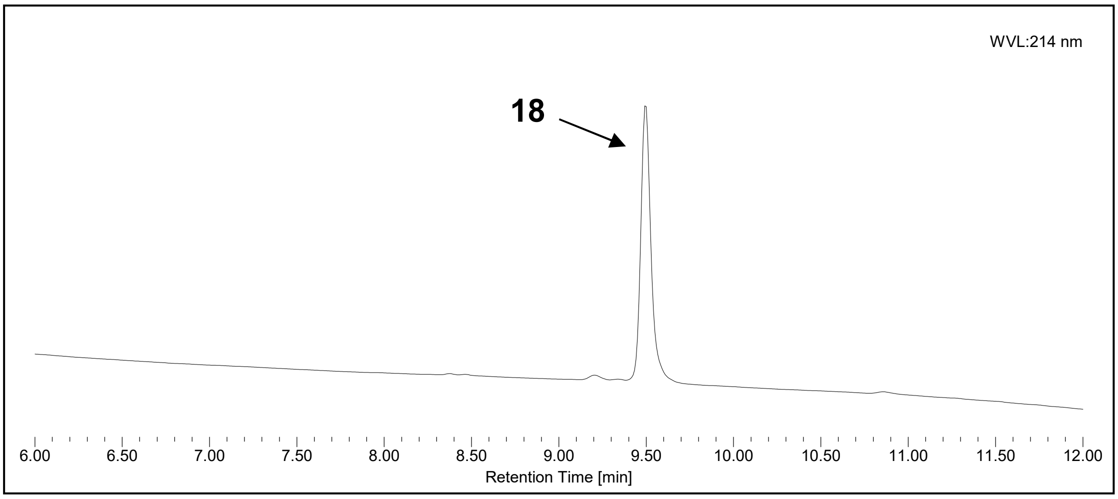
WVL:214 nm
18
6.00
6.50
7.00
7.50
8.00
8.50
9.00
9.50
10.00
10.50
11.00
11.50
12.00
Retention Time [min]

HPLC chromatograms for cyclisations of peptides (12) and (13) and selective deprotection of peptide (13)

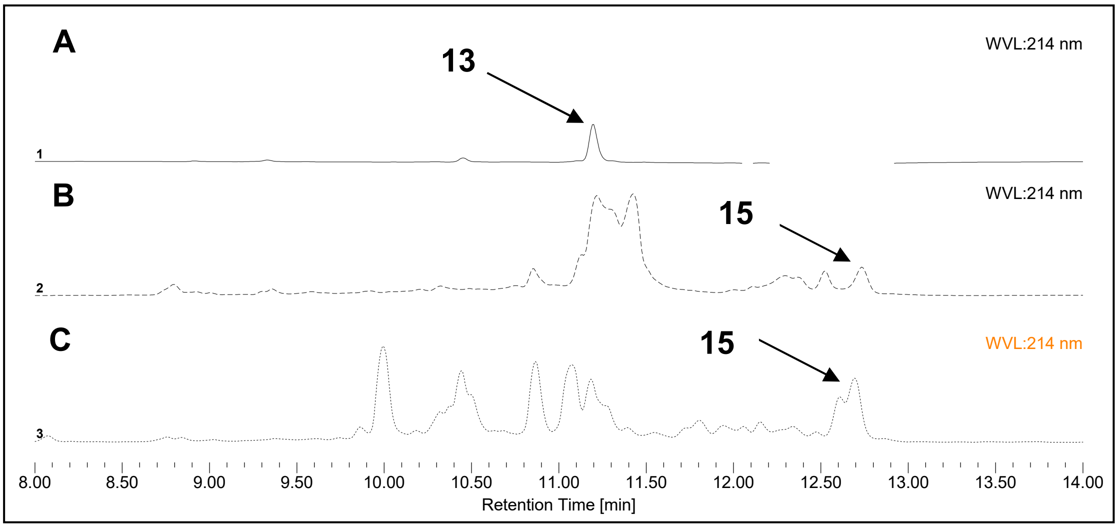

Figure S1. HPLC chromatograms for the cyclization of **13**.

A. Linear fOHOrn peptide **13.**
B. Crude material after treatment of **13** with HATU (10 eq), DIEA (10 eq) in DMF for 14 h. Cyclic peptide **15** is highlighted.
C. Crude material after treatment of **13** with HATU (10 eq), DIEA (10 eq) in DMF for 48 h. Cyclic peptide **14** is highlighted.

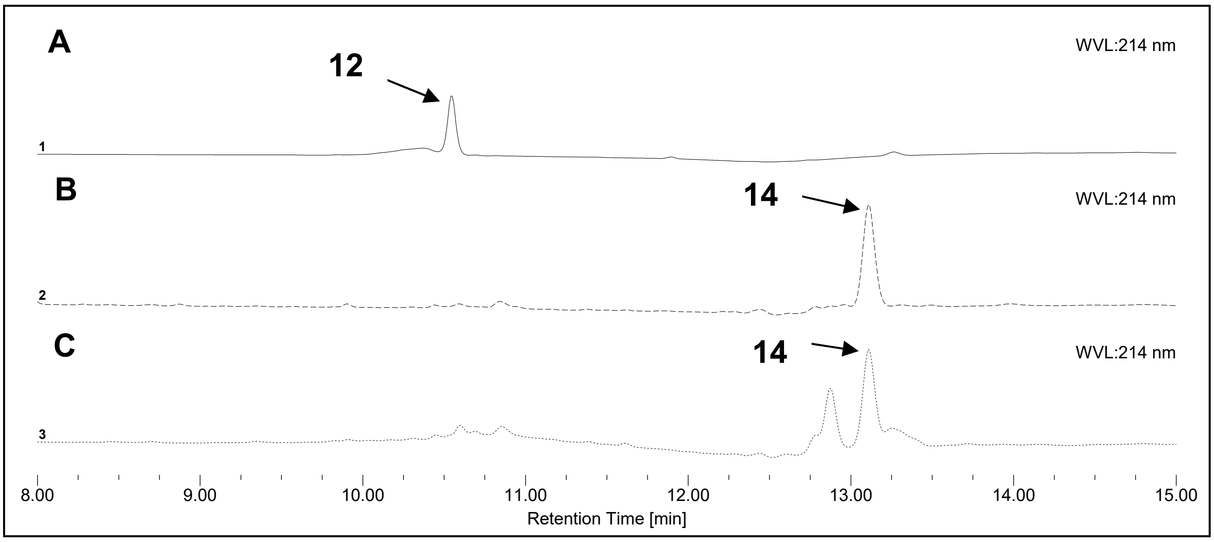

Figure S2. HPLC chromatograms for the cyclization of **12**.

A. Linear Orn(Boc) peptide **12.**
B. Crude material after treatment of **12** with PyBOP (10 eq), DIEA (10 eq) in DMF for 2 h. Cyclic peptide **14** is highlighted.
C. Crude material after treatment of **12** with HATU (10 eq), DIEA (10 eq) in DMF for 2 h. Cyclic peptide **14** is highlighted.

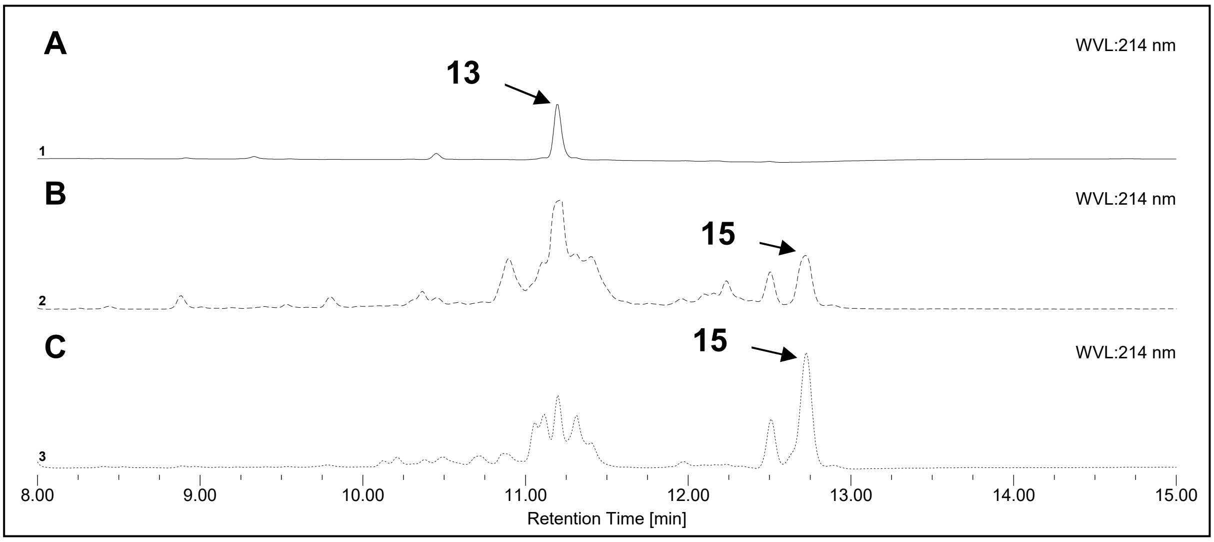

Figure S3. HPLC chromatograms for the cyclization of **13**.

A. Linear fOHOrn peptide **13.**
B. Crude material after treatment of **13** with PyBOP (10 eq), DIEA (10 eq) in DMF for 2 h. Cyclic peptide **15** is highlighted.
C. Crude material after treatment of **13** with PyBOP (1.2 eq), DIEA (2 eq) in DMF for 2 h. Cyclic peptide **15** is highlighted.

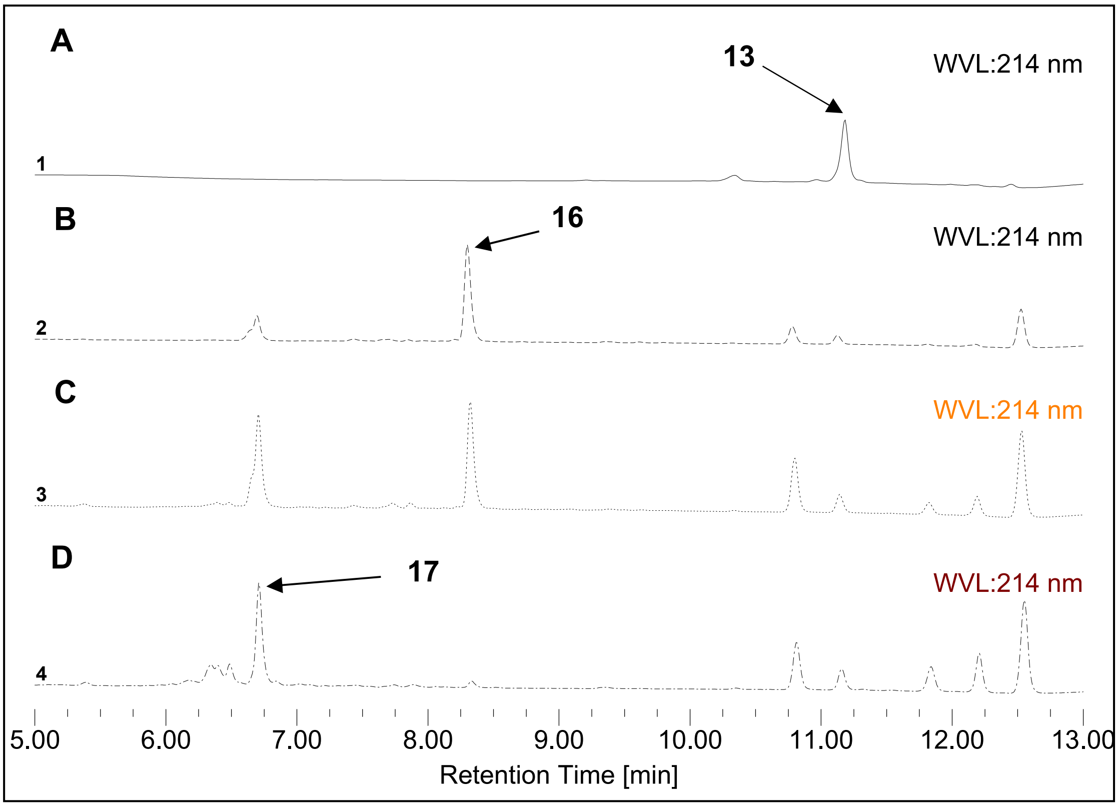

Figure S4. HPLC chromatograms for the treatment of **13** with 0.1M HCl in HFIP.

A. Linear fOHOrn peptide **13.**
B. Reaction progress after 10 min showing formation of partially side chain deprotected peptide **16.**
C. Reaction progress after 30 min showing both **16** and fully side chain deprotected peptide **17.**
D. Reaction progress after 60 min showing disappearance of **16.**

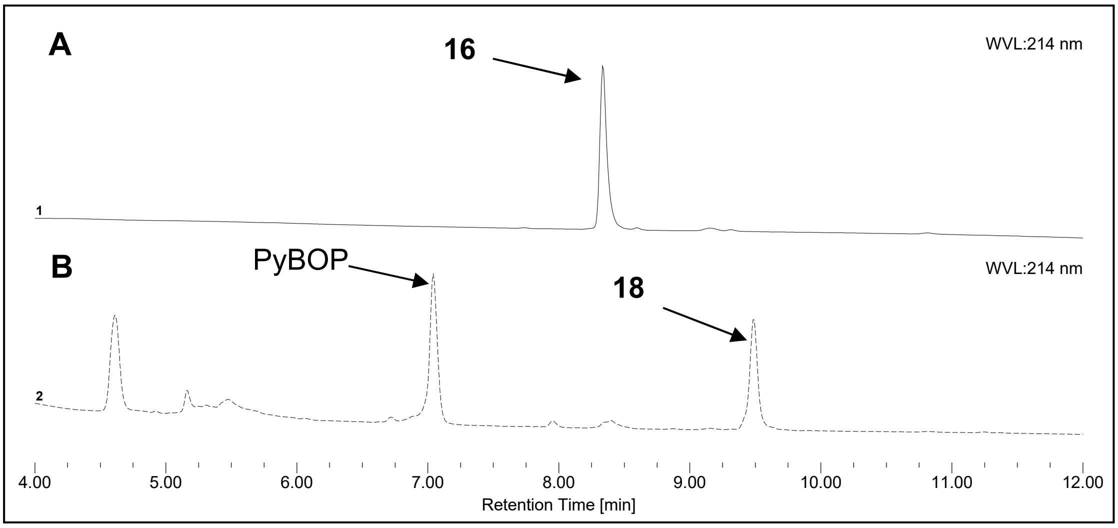

Figure S5. HPLC chromatograms for the cyclization of **16**.

A. Partially side chain deprotected linear peptide **16.**
B. Reaction progress after 30 min showing the formation of cyclic peptide **18.**

Reference

34. Bialy, L.; Díaz-Móchon, J.J.; Specker, E.; Keinicke, L.; Bradley, M. Dde-protected PNA monomers, orthogonal to Fmoc, for the synthesis of PNA-peptide conjugates. *Tetrahedron* **2005**, *61*, 8295-8305.
